# Supplementary material for: Commercial hatchery practices have long-lasting effects on laying hens’ spatial behaviour and health
Source: PLoS One. 2023 Dec 20;18(12):e0295560. doi: 10.1371/journal.pone.0295560 (PMC10732460; doi:10.1371/journal.pone.0295560)
Supplement: S1 Text — (PDF) [file pone.0295560.s001.pdf]

S1 Text. More/less explorer class.

This was copied from the Supplementary materials in [1]:

> "We installed one raised platform with blue dye per pen when chicks were one day old, so that more explorer chicks would have blue feet. We planned to change the blue dye with a violet dye once at least 30 chicks could be classified as "more explorer", in each of the four rearing pens (600 hens / pen). By testing the dye previously on dead hens, we knew that feet colored by the blue dye and then by the violet dye, would have been distinguishable to feet colored by the violet dye only. However, there was not enough more explorer chicks within a suitable timeframe (< 5 days) to allow for the intermediate class, which we believe was needed to reliably differentiate the two classes of interest (more and less explorers). Furthermore, we added dye diluted in water approximately every two hours to prevent the dye from drying out, but we noticed on some occasions that the dye had already dried out. Therefore, class remained unvalidated, and we included it in the variable in our model only to control as we used it to help select individuals."

References

1. Montalcini CM, Petelle MB, Toscano MJ. 2023 Commercial laying hens exhibit long-term consistent individual differences and behavioural syndromes in spatial traits. *R Soc Open Sci* **10**. (doi:10.1098/RSOS.230043)
