# Supplementary figures and images for: Commercial hatchery practices have long-lasting effects on laying hens’ spatial behaviour and health
Source: PLoS One. 2023 Dec 20;18(12):e0295560. doi: 10.1371/journal.pone.0295560 (PMC10732460; doi:10.1371/journal.pone.0295560)

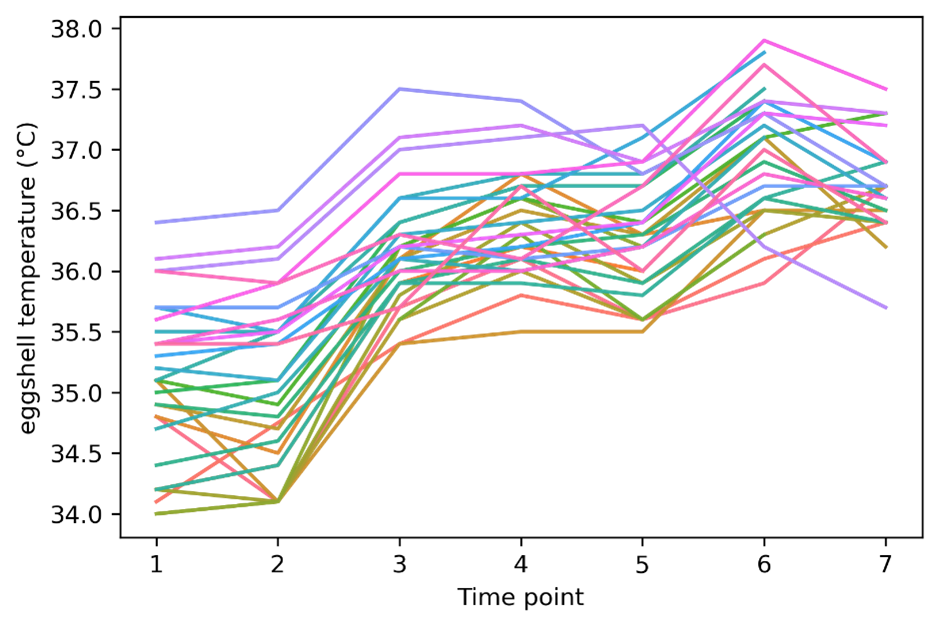

Supplement: S1 Fig — We monitored 30 OFH eggs every six hours, for a total of 7 timestamps, until a significant proportion of the chicks hatched. (PNG) [file pone.0295560.s002.png]

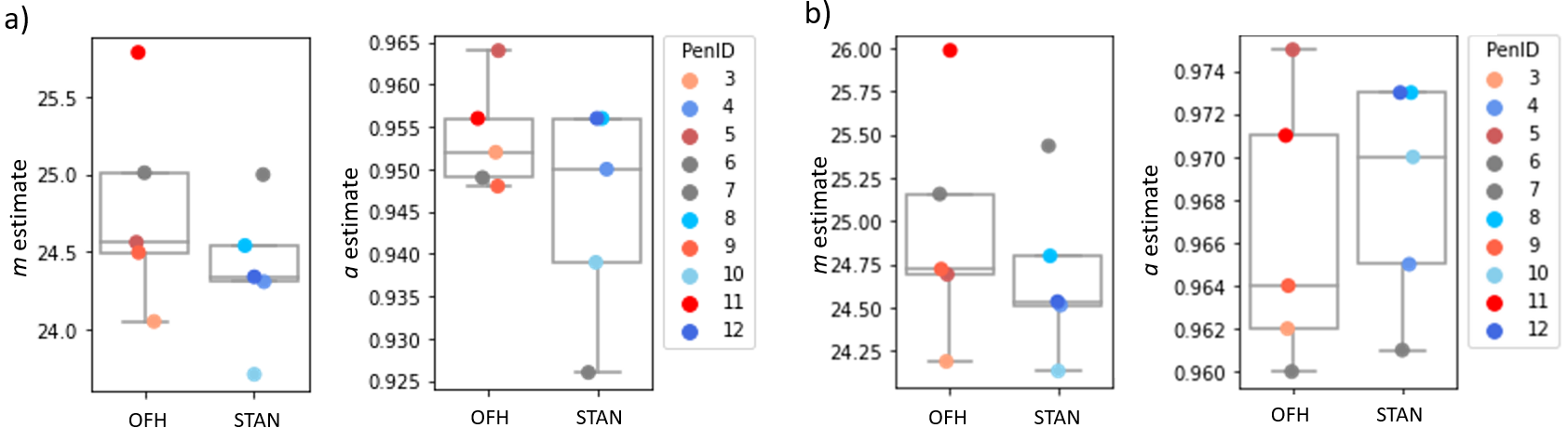

Supplement: S2 Fig — Parameter a may be viewed as an indication of the level at which egg production stabilise. Parameter m as an indication of the time point at the inflection point of the curve. These estimates of the curves fitting the first 60 days and the full period in the laying barn are given in (b) and (d), respectively. (PNG) [file pone.0295560.s003.png]
