# Supplementary material for: Commercial hatchery practices have long-lasting effects on laying hens’ spatial behaviour and health
Source: PLoS One. 2023 Dec 20;18(12):e0295560. doi: 10.1371/journal.pone.0295560 (PMC10732460; doi:10.1371/journal.pone.0295560)
Supplement: S1 Table — (PDF) [file pone.0295560.s004.pdf]

Time point	Pen : 1.1.1	Pen : 1.1.2	Pen : 1.1.3
1 june 13h	1	0	0
1 june 18h	1	0	0
1 june-2 june 00h	1	0	0
2 june 6h	2	1	1
2 june 12h	8	26	16
2 june 18h	-	-	-
2 june-3 june 00h	~15%	~40%	~25%
3 june 6h	~75%	~80%	~80%
3 june 12h	>80%	>80%	>80%
3 june 18h	>80%	>80%	>80%
3 june-4 june 00h	>80%	>80%	>80%
4 june 6h	>80%	>80%	>80%

S1 Table. Hatching rate over time for the OFH chicks.
