# Supplementary material for: Commercial hatchery practices have long-lasting effects on laying hens’ spatial behaviour and health
Source: PLoS One. 2023 Dec 20;18(12):e0295560. doi: 10.1371/journal.pone.0295560 (PMC10732460; doi:10.1371/journal.pone.0295560)
Supplement: S2 Table — (PDF) [file pone.0295560.s005.pdf]

	Feather damage		Body mass (g)		KBF severity	
**DOA**	OFH	STAN	OFH	STAN	OFH	STAN
**7**			65.54<br>[64.41, 66.67]	64.70<br>[63.58, 65.82]		
**118**			1170.91<br>[1158.12, 1183.71]	1161.00<br>[1148.06, 1173.94]		
**173**			1686.78<br>[1670.18, 1703.39]	1673.76<br>[1656.97, 1690.54]		
**215**			1792.27<br>[1772.30, 1812.25]	1777.06<br>[1756.72, 1797.40]	17.70<br>[14.70, 20.80]	13.20<br>[10.1, 16.3]
**243**	9.44<br>[6.80, 12.10]	8.49<br>[5.84, 11.10]	1824.61<br>[1795.20, 1854.02]	1801.59<br>[1771.95, 1831.23	21.30<br>[18.20, 24.30]	16.70<br>[13.70, 19.80]
**313**	17.43<br>[14.79, 20.10]	16.48<br>[13.82, 19.10]	1884.65<br>[1848.58, 1920.71]	1856.46<br>[1820.12, 1892.80]	29.60<br>[26.60, 32.70]	25.10<br>[22.00, 28.20]
**417**	34.12<br>[31.46, 36.80]	33.17<br>[30.50, 35.80]	1905.84<br>[1877.94, 1933.74]	1884.41<br>[1856.34, 1912.49]	39.50<br>[36.40 42.60]	35.00<br>[31.90, 38.10]

S2 Table. **Estimated marginal means and 95% confidence intervals for welfare indicators**.
