# Supplementary material for: Commercial hatchery practices have long-lasting effects on laying hens’ spatial behaviour and health
Source: PLoS One. 2023 Dec 20;18(12):e0295560. doi: 10.1371/journal.pone.0295560 (PMC10732460; doi:10.1371/journal.pone.0295560)
Supplement: S3 Table — (PDF) [file pone.0295560.s006.pdf]

	severity		FeatherDamage		weight_norm	
Predictors	*Estimates*	*p*	*Estimates*	*p*	*Estimates*	*p*
(Intercept)	13.29 *** (10.15 – 16.43)	**<0.001**	8.88 *** (6.53 – 11.24)	**<0.001**	0.51 *** (0.47 – 0.54)	**<0.001**
CLASS [LEXP]	0.86 (-4.08 – 5.80)	0.732	-1.97 (-5.10 – 1.16)	0.217	-0.01 (-0.07 – 0.04)	0.677
CLASS [MEXP]	-1.17 (-5.67 – 3.33)	0.610	0.79 (-2.07 – 3.64)	0.588	0.01 (-0.04 – 0.06)	0.677
date2021-02-01	3.56 ** (1.39 – 5.73)	**0.001**			-0.05 *** (-0.08 – -0.02)	**<0.001**
date2021-04-12	11.92 *** (9.71 – 14.14)	**<0.001**	7.99 *** (6.48 – 9.50)	**<0.001**	0.08 *** (0.06 – 0.11)	**<0.001**
date2021-07-25	21.77 *** (19.48 – 24.07)	**<0.001**	24.68 *** (23.10 – 26.25)	**<0.001**	-0.05 *** (-0.08 – -0.03)	**<0.001**
Treatment [OFH]	4.53 * (0.99 – 8.07)	**0.012**	0.95 (-1.95 – 3.85)	0.519	0.03 (-0.01 – 0.06)	0.166
date2020-09-29					-0.01 (-0.03 – 0.02)	0.584
date2020-11-23					0.05 *** (0.03 – 0.08)	**<0.001**
date2021-01-04					-0.06 *** (-0.09 – -0.03)	**<0.001**
**Random Effects**						
σ^2	101.10		49.72		0.01	
τ_{00}	125.66 $_{HenID}$		43.07 $_{HenID:PenID}$		0.02 $_{HenID}$	
			1.73 $_{PenID}$			
ICC	0.55		0.47		0.58	
N	194 $_{HenID}$		194 $_{HenID}$		231 $_{HenID}$	
			8 $_{PenID}$			
Observations	669		506		1121	
Marginal R^2 / Conditional R^2	0.244 / 0.663		0.522 / 0.749		0.090 / 0.619	

* *p<0.05* ** *p<0.01* *** *p<0.001*

S3 Table. Model outputs for welfare indicators.
