# Supplementary material for: Commercial hatchery practices have long-lasting effects on laying hens’ spatial behaviour and health
Source: PLoS One. 2023 Dec 20;18(12):e0295560. doi: 10.1371/journal.pone.0295560 (PMC10732460; doi:10.1371/journal.pone.0295560)
Supplement: S4 Table — (PDF) [file pone.0295560.s007.pdf]

month	proportion of time spent in top tier (exp())	proportion of time spent in litter floor	proportion of time spent in nestbox tier (exp())	WG presence (exp())	vertical travelled distance	mid-nestbox tier usage (exp())
1	**1.49 [1.12, 2.05], p=0.01**			0.50 [0.17, 1.22], p=0.13		
2	**1.67 [1.14, 2.35], p=0.00**	**-0.07 [-0.11, -0.02], p=0.01**	0.96 [0.87, 1.08], p=0.50	0.98 [0.40, 2.37], p=0.96	**-10.62 [-16.13, -4.35], p=0.00**	**0.96 [0.92, 1.00], p=0.02**
3	1.38 [0.90, 2.06], p=0.12	**-0.05 [-0.11, -0.00], p=0.04**	0.99 [0.87, 1.12], p=0.93	1.03 [0.35, 3.10], p=0.95	**-9.19 [-16.13, -2.50], p=0.01**	0.99 [0.95, 1.04], p=0.77
4	1.26 [0.86, 2.02], p=0.26	-0.04 [-0.09, 0.01], p=0.18	0.99 [0.87, 1.13], p=0.86	0.67 [0.23, 1.80], p=0.46	-6.53 [-14.38, 1.07], p=0.10	1.02 [0.97, 1.08], p=0.36
5	1.44 [0.89, 2.27], p=0.10	-0.04 [-0.09, 0.01], p=0.11	1.04 [0.90, 1.23], p=0.64	0.55 [0.17, 1.87], p=0.36	-4.08 [-11.11, 3.22], p=0.30	1.02 [0.96, 1.09], p=0.60
6	1.20 [0.82, 1.83], p=0.28	-0.04 [-0.09, 0.01], p=0.14	1.06 [0.91, 1.24], p=0.48	0.57 [0.24, 1.34], p=0.19	-3.87 [-11.33, 3.05], p=0.31	1.01 [0.97, 1.06], p=0.60
7	1.30 [0.90, 1.90], p=0.20	-0.03 [-0.08, 0.02], p=0.28	1.00 [0.89, 1.14], p=1.00	0.73 [0.33, 1.63], p=0.47	-2.90 [-9.40, 4.00], p=0.41	1.00 [0.95, 1.06], p=0.90
8	1.01 [0.63, 1.46], p=0.97	0.00 [-0.05, 0.06], p=0.90	0.99 [0.87, 1.12], p=0.88	1.05 [0.41, 3.00], p=0.88	1.22 [-5.44, 8.13], p=0.73	1.01 [0.96, 1.07], p=0.64
9	1.20 [0.77, 1.84], p=0.40	-0.01 [-0.06, 0.04], p=0.76	0.94 [0.83, 1.05], p=0.29	0.74 [0.25, 1.78], p=0.55	-0.69 [-7.22, 5.49], p=0.82	0.99 [0.94, 1.04], p=0.64
10	1.03 [0.70, 1.61], p=0.89	-0.01 [-0.05, 0.04], p=0.82	0.94 [0.85, 1.04], p=0.27	0.13 [0.01, 1.07], p=0.07	-0.38 [-5.46, 5.55], p=0.93	1.00 [0.95, 1.05], p=0.97

S4 Table. Bootstrapped estimates and p-values for the model fitting spatial behaviours.
