# Supplementary material for: Commercial hatchery practices have long-lasting effects on laying hens’ spatial behaviour and health
Source: PLoS One. 2023 Dec 20;18(12):e0295560. doi: 10.1371/journal.pone.0295560 (PMC10732460; doi:10.1371/journal.pone.0295560)
Supplement: Attachment — Submitted filename: PONE-D-23-28828.pdf [file pone.0295560.s008.pdf]

PLOS ONE

Commercial hatchery practices have long-lasting effects on laying hens' spatial behaviour and health

--Manuscript Draft--

**Manuscript Number:**	PONE-D-23-28828
**Article Type:**	Research Article
**Full Title:**	Commercial hatchery practices have long-lasting effects on laying hens' spatial behaviour and health
**Short Title:**	Commercial hatchery practices and animal welfare
**Corresponding Author:**	Camille M. Montalcini<br>University of Bern Vetsuisse Faculty: Universitat Bern Vetsuisse Fakultat<br>Bern, SWITZERLAND
**Keywords:**	early-life stress; on-farm hatch; animal welfare; spatial behaviour; Gallus gallus domesticus
**Abstract:**	The commercial hatchery process is globally standardized and exposes billions of day-old layer chicks to stress every year. By alleviating this early stress, on-farm hatching is thought to improve animal welfare, yet little is known about its effects throughout production. This study compared the welfare and spatial behaviours during the laying period of hens hatched in an on-farm environment (OFH) to those hatched in a commercial hatchery and transferred at one day-old to a rearing barn (STAN). Specifically, we assessed how the transfer to the laying barn at 17 weeks of age, a similar stressor encountered by STAN hens early in life, affected space use and health, and determined whether effects aligned more with the 'silver-spoon' or 'environmental matching' hypothesis. We found that for the first three months post-transfer into the laying barn, OFH hens, on average, transitioned less between the aviary's tiers and spent less time on the littered floor. Because OHF hens became behaviourally more similar to STAN hens over time, these results suggest that OFH hens required a prolonged period to establish their daily behavioural patterns. Furthermore, OFH hens had more severe keel bone fractures throughout the laying period but similar feather damage and body mass to STAN hens. No differences were observed in overall egg-production. These findings support the environmental matching hypothesis and suggest that early-life stressors may have prepared hens for later-life stressors, underscoring the importance of both early-life and adult environments in enhancing animal welfare throughout production.
**Order of Authors:**	Camille M. Montalcini
	Matthew B. Petelle
	Michael J. Toscano
**Additional Information:**	
**Question**	**Response**
**Financial Disclosure**<br><br>Enter a financial disclosure statement that describes the sources of funding for the work included in this submission. Review the submission guidelines for detailed requirements. View published research articles from *PLOS ONE* for specific examples.<br><br>This statement is required for submission and **will appear in the published article** if	M.J.T. received funding from the Swiss National Science Foundation (grant number 310030_189056) and the European Union's Horizon 2020 research and innovation programme (grant agreement N°101000236). The document reflects only the author's view and the European Union's Horizon 2020 research, and innovation programme is not responsible for any use that may be made of the information it contains. This project is part of EuroFAANG (https://eurofaang.eu). The funders had no role in study design, data collection and analysis, decision to publish, or preparation of the manuscript.

Powered by Editorial Manager® and ProduXion Manager® from Aries Systems Corporation

the submission is accepted. Please make sure it is accurate.<br><br>**Unfunded studies**<br>Enter: *The author(s) received no specific funding for this work.*<br><br>**Funded studies**<br>Enter a statement with the following details:<br>• Initials of the authors who received each award<br>• Grant numbers awarded to each author<br>• The full name of each funder<br>• URL of each funder website<br>• Did the sponsors or funders play any role in the study design, data collection and analysis, decision to publish, or preparation of the manuscript?<br>• **NO** - Include this sentence at the end of your statement: *The funders had no role in study design, data collection and analysis, decision to publish, or preparation of the manuscript.*<br>• **YES** - Specify the role(s) played.<br><br>* typeset	
**Competing Interests**<br><br>Use the instructions below to enter a competing interest statement for this submission. On behalf of all authors, disclose any competing interests that could be perceived to bias this work—acknowledging all financial support and any other relevant financial or non-financial competing interests.<br><br>This statement is required for submission and **will appear in the published article** if the submission is accepted. Please make sure it is accurate and that any funding sources listed in your Funding Information later in the submission form are also declared in your Financial Disclosure statement.<br><br>View published research articles from *PLOS ONE* for specific examples.	The authors have declared that no competing interests exist.

**NO authors have competing interests**<br><br>Enter: *The authors have declared that no competing interests exist.*<br><br>**Authors with competing interests**<br><br>Enter competing interest details beginning with this statement:<br><br>*I have read the journal's policy and the authors of this manuscript have the following competing interests: [insert competing interests here]*<br><br>* typeset	
**Ethics Statement**<br><br>Enter an ethics statement for this submission. This statement is required if the study involved:<br><br>• Human participants<br>• Human specimens or tissue<br>• Vertebrate animals or cephalopods<br>• Vertebrate embryos or tissues<br>• Field research<br><br>Write "N/A" if the submission does not require an ethics statement.<br><br>General guidance is provided below. Consult the submission guidelines for detailed instructions. **Make sure that all information entered here is included in the Methods section of the manuscript.**	The research was conducted in accordance with the cantonal and federal regulations for the ethical treatment of experimentally used animals. All procedures were approved by the Bern Cantonal Veterinary Office (BE-45/20).

Powered by Editorial Manager® and ProduXion Manager® from Aries Systems Corporation

**Format for specific study types**<br><br>**Human Subject Research (involving human participants and/or tissue)**<br>• Give the name of the institutional review board or ethics committee that approved the study<br>• Include the approval number and/or a statement indicating approval of this research<br>• Indicate the form of consent obtained (written/oral) or the reason that consent was not obtained (e.g. the data were analyzed anonymously)<br><br>**Animal Research (involving vertebrate animals, embryos or tissues)**<br>• Provide the name of the Institutional Animal Care and Use Committee (IACUC) or other relevant ethics board that reviewed the study protocol, and indicate whether they approved this research or granted a formal waiver of ethical approval<br>• Include an approval number if one was obtained<br>• If the study involved *non-human primates*, add *additional details* about animal welfare and steps taken to ameliorate suffering<br>• If anesthesia, euthanasia, or any kind of animal sacrifice is part of the study, include briefly which substances and/or methods were applied<br><br>**Field Research**<br><br>Include the following details if this study involves the collection of plant, animal, or other materials from a natural setting:<br>• Field permit number<br>• Name of the institution or relevant body that granted permission	
**Data Availability**<br><br>Authors are required to make all data underlying the findings described fully available, without restriction, and from the time of publication. PLOS allows rare exceptions to address legal and ethical concerns. See the PLOS Data Policy and FAQ for detailed information.	Yes - all data are fully available without restriction

A Data Availability Statement describing where the data can be found is required at submission. Your answers to this question constitute the Data Availability Statement and **will be published in the article**, if accepted.<br><br>**Important:** Stating 'data available on request from the author' is not sufficient. If your data are only available upon request, select 'No' for the first question and explain your exceptional situation in the text box.<br><br>Do the authors confirm that all data underlying the findings described in their manuscript are fully available without restriction?	
**Describe where the data may be found in full sentences. If you are copying our sample text, replace any instances of XXX with the appropriate details.**<br><br>• If the data are **held or will be held in a public repository**, include URLs, accession numbers or DOIs. If this information will only be available after acceptance, indicate this by ticking the box below. For example: *All XXX files are available from the XXX database (accession number(s) XXX, XXX.)*.<br>• If the data are all contained **within the manuscript and/or Supporting Information files**, enter the following: *All relevant data are within the manuscript and its Supporting Information files.*<br>• If neither of these applies but you are able to provide **details of access elsewhere**, with or without limitations, please do so. For example:<br><br>*Data cannot be shared publicly because of [XXX]. Data are available from the XXX Institutional Data Access / Ethics Committee (contact via XXX) for researchers who meet the criteria for access to confidential data.*<br><br>*The data underlying the results presented in the study are available from (include the name of the third party*	The data and code for this study are available on https://doi.org/10.17605/OSF.IO/5WQGD

and contact information or URL).<br>• This text is appropriate if the data are owned by a third party and authors do not have permission to share the data.<br><br>* typeset	
Additional data availability information:	

manuscript Click here to access/download;Manuscript;Manuscript.docx

Commercial hatchery practices have long-lasting effects on laying hens' spatial behaviour and health

Camille M. Montalcini[1,2], Matthew B. Petelle[1], and Michael J. Toscano[1]

1 ZTHZ, Division of Animal Welfare, VPH Institute, University of Bern, 3052 Zollikofen, Switzerland
2 Graduate School of Cellular and Biomedical Sciences, University of Bern, 3012 Bern, Switzerland
*Corresponding author: camille.montalcini@unibe.ch

Abstract

The commercial hatchery process is globally standardized and exposes billions of day-old layer chicks to stress every year. By alleviating this early stress, on-farm hatching is thought to improve animal welfare, yet little is known about its effects throughout production. This study compared the welfare and spatial behaviours during the laying period of hens hatched in an on-farm environment (OFH) to those hatched in a commercial hatchery and transferred at one day-old to a rearing barn (STAN). Specifically, we assessed how the transfer to the laying barn at 17 weeks of age, a similar stressor encountered by STAN hens early in life, affected space use and health, and determined whether effects aligned more with the 'silver-spoon' or 'environmental matching' hypothesis. We found that for the first three months post-transfer into the laying barn, OFH hens, on average, transitioned less between the aviary's tiers and spent less time on the littered floor. Because OHF hens became behaviourally more similar to STAN hens over time, these results suggest that OFH hens required a prolonged period to establish their daily behavioural patterns. Furthermore, OFH hens had more severe keel bone fractures throughout the laying period but similar feather damage and body mass to STAN hens. No differences were observed in overall egg-production. These findings support the environmental matching hypothesis and suggest that early-life stressors may have prepared hens for later-life stressors, underscoring the importance of both early-life and adult environments in enhancing animal welfare throughout production.

Keywords: early-life stress, on-farm hatch, animal welfare, spatial behaviour, *Gallus gallus domesticus*

Introduction

Every year, billions [1] of day-old layer chicks are exposed to stress [2] because of the globally standardized commercial hatchery process [3]. Chicks in hatcheries, are subjected to loud noise during incubation (~90dB), hatched in darkness, prevented from accessing feed, water, or litter, and subjected to sexing, vaccination, and transportation to rearing farms at one day of age [2]. This early exposure to stress can be alleviated by hatching chicks on farm, where chicks are transported before hatching and have direct access to feed, water and litter.

Exposure to less stressful environmental conditions during development may have a positive impact on various aspects of individual fitness later on, a phenomenon called "silver-spoon" effect [4,5]. The silver-

spoon hypothesis suggests that individuals who experience good environmental conditions during development, such as characterized by reduced stressors or abundant nutritional availability, may be able to cope better when confronted with adversity later in life. Accordingly, it is not surprising that on-farm hatching has been shown to improve the well-being of broilers and laying hens later in life, in particular throughout development. Specifically, this practice has been shown to reduce total mortality [6] and footpad dermatitis [7,8] of broilers, and increase body mass [3,9,10] and reduce feather damage, comb injuries, and corticosterone reactivity during restraint [2] of laying hens.

Alternatively, by experiencing adverse environments during development, one may be prepared or adapted to handle similar adversities in the future. The "environmental matching" hypothesis suggests that environmental conditions in early life shape an individual phenotype via developmental plasticity [11], so that an individual is adapted to similar environmental conditions experienced earlier in life [12,13]. Therefore, chicks that hatched in a commercial hatchery and subsequently transported on farm could have a phenotype more adapted to aversive environments, such as transportation to a new environment, than chicks hatched on-farm. Thus, the benefit of a less aversive early life environment would depend on the environmental conditions experienced later in life [12–14]. However, studies evaluating the effect of the commercial hatchery process on adult laying hens in commercial settings are scarce, limiting our understanding of their long-term effects on hen welfare. This is especially true for health issues that predominantly arise during adulthood or may worsen as hens age, such as feather damage [15,16] or keel bone fractures (KBF) [17,18]. Thus, a long-term approach is necessary to understand whether on-farm hatching improves hen welfare throughout production, or whether its relative benefits are eventually offset by later stressors that they are unable to manage.

In this study, we compared the severity of KBF, feather damage, body mass and spatial behaviours of laying hens hatched on-farm (OFH) to those hatched in a commercial hatchery and transferred at one day-old to the farm (STAN). Our goal was to determine if, and for how long, these two different environments experienced at one day of age could account for variations in animal welfare and behaviour during the laying period. We assessed how the transfer from the rearing to the laying barn, a similar stressor encountered by day-old STAN chicks, affected space-use and movement behaviours, as well as welfare indicators. We aimed to determine whether the observed effects aligned more with the 'silver-spoon' or 'environmental matching' hypothesis. The former hypothesis would be supported if OFH hens would display overall greater welfare compared to STAN hens, while the latter hypothesis would be supported if OFH would display overall worse welfare conditions. Better welfare could be here manifested via less severe KBF, reduced feather damage, and greater time spent in the littered floor and the winter garden. These areas provide enhanced opportunities for the expression of natural behaviours such as locomotion, exploring, foraging, scratching, and dust bathing, which are important for laying hen welfare [19,20].

Materials and Methods

Ethical note

The research was conducted in accordance with the cantonal and federal regulations for the ethical treatment of experimentally used animals. All procedures were approved by the Bern Cantonal Veterinary Office (BE-45/20).

Study design

All Dekalb white chicks (*Gallus gallus domesticus*) originated from the same parent flock and began incubation off-site using standard hatchery practices. At 18 days of development, three days before hatching, 3,300 eggs were arbitrarily chosen as part of the on-farm hatch (OFH) treatment and transported to a commercial rearing barn at the Aviforum facilities in Switzerland. The eggs were transported in a commercial vehicle for less than 1.5 hours, which maintained a stable environmental temperature at an average of 36.4 °C. Eggs were positioned in HatchTech Setter Trays 15 cm above the littered floor, where feed, water, and litter were available. We monitored specific eggs every six hours throughout the hatching process to ensure maintenance of environmental conditions so that eggshell temperature remained between 35-38 °C and ambient relative humidity above 30%.

At one day of age (DOA), OFH hens were manually sexed and vaccinated. On the same day, 1,200 chicks from the commercial hatchery were transported to the rearing barn as part of the STAN treatment. This transportation took place in a commercial vehicle over a duration of eight hours, during which a consistent environmental temperature around 28 °C was maintained. Unlike the OFH chicks, STAN chicks - in addition to being transported to a new housing - hatched in darkness and were deprived direct access to food, water, or litter after hatching until arrival at the rearing barn. Similar to OFH chicks, STAN chicks were manually sexed and received a vaccination in the rearing barn alongside the OFH chicks. STAN chicks were used to populate two rearing pens and OFH chicks were used to populate the other two pens (600 hens/pen). Males and surplus females were returned to the hatchery for humane disposal. At seven DOA all chicks were classified into a more/less explorer class. We did not use the class as an exploratory behaviour as the measurement could not be validated (Supplementary text 1), though we controlled for the class in subsequent analysis. Simultaneously, 160 focal birds (40 hens/rearing pen) were selected from the 2’400 chicks. Of the 160 focal birds, 80 focal birds were classified as MEXP or LEXP (40 / class), while 80 were selected as a representative sample and used for another study that collected brain tissues throughout the laying period.

At 119 DOA, all hens were caught, put into a crate, and transported to one of eight laying pens on the same site (225 hens/pens, including 20 focal hens, four pens/treatment). The laying barn contained a quasi-commercial multi-tier aviary system (Bolegg Terrace separated into 20 pens by grids illustrated in

Supplementary Figure S2; indoor length x width x height until the top tier grid floor: 7 x 2.3 x 2.69 m ; previously described [21]) and an outside covered winter garden (WG ; $9.32\ m^2$) accessible by pop holes from 10:00 h to 16:00 h on most days. Animal density was 8.1 hens per square-meter of permanent accessible area (225 hens/27.92 m^2). On the day of transfer to the laying barn the 160 focal hens were assigned a tracking device to continuously register their transitions across the indoor aviary levels and the winter garden (WG) until near the end of production (tracking period: September 2020 – July 2021). At five time points during the laying period (DOA: 127, 173, 243, 313, and 418), 16 randomly selected focal hens were killed (eight hens/treatment) to collect brain tissues as part of a separate study. Each of these time points also included welfare assessment (described in the below section), except for DOA 127, which was replaced by DOA 215 to capture more variation in animal welfare. For each hen killed, another hen from the same pen was arbitrarily selected to continuously track the same number of hens, for a total of 227 hens used in the study.

Welfare indicators

Welfare assessment included feather damage, radiographs for KBF, and body mass (digital scale in grams). During the welfare assessment, the observers were blinded to the treatment, laying pen identity, and hen class, and shown reliable in a previous effort for both feather damage and KBF severity scores [22]. The feather damage score (continuous, 0-100) was assigned using the photographs of white laying hens which we rescaled to 0-100 and took the complement to 100 so that higher scores are indicative of poorer welfare (score 1: approx. 100 – 76 depending on the extent of damage; score 2: approx. 75 – 51 ; etc.) for each body part [23]. More precisely, we assigned a score of the breast, tail, and neck, but not the back and wing feathers as these could not be reliably assessed because of the backpack containing the tracking tag (described below). We then averaged these to get an overall individual feather damage score. We assessed KBF severity (continuous, 0-100) based on the latero-lateral radiographs using the scoring methodology described by Rufener et al. [24], where the score is described as an indicator of the total amount of keel bone affected by fractures. We excluded the first timepoint of KBF severity and feather damage, as there was little variation, with both having a median score of zero.

Spatial behaviours

We tracked individuals' transitions across five zones: the four different levels of the aviary (top tier, nestbox tier, lower tier, and littered floor) and the outside covered WG. We used a low-frequency tracking system with active tags (mass: 28.1 g) enclosed in a backpack mounted on the back of the hens (see Montalcini et al. (2021) [25] for the validation and description). Tracking data were collected from the first full day in the laying barn (DOA 119) until near the end of production (DOA 416). We excluded days with known disturbances (e.g., vaccinations or welfare assessments) and those with known tracking system malfunctions (e.g., low battery level). Subsequent analysis involved a period of 297 days of tracking, during which hens had on average 169

days tracked, with a minimum of three days tracked and a maximum of 250 days, involving a total of 227 hens and 38,303 hen-days observations.

We characterized the daily movement and space-use behaviours of each hen with six behaviours expressed while artificial light was provided. We used the (i) vertical travelled distance, defined as the total number of indoor tiers crossed, to account for the level of vertical movement. We used the proportion of the indoor time spent on the (ii) top tier, (iii) nestbox tier, and (iv) littered floor to account for indoor space-use behaviours, and (v) WG presence (yes/no) to account for the outdoor space-use behaviour. Finally, because the nestbox tier is of particular interest within commercial settings, we also used the (vi) time when a hen reached half of its nestbox tier duration, accounting only for hours where hens are expected to lay, that is between 2 and 8 hour, hereafter referred to as the nestbox tier timing.

Egg-production data

We used pen-level production data to assess whether treatment could account for variation in egg-production. Throughout the laying period, eggs laid inside the nestboxes were collected consistently at the same time every day and counted at the pen-level. In addition to the four pens per treatment group (STAN and OFH) with focal animals, we had access to two additional pens without focal hens but containing 205 animals from one treatment group with an additional 20 Lohmann LSL hens that hatched in the hatchery. In subsequent analysis, we used the daily average number of eggs per live hen in each pen, considering all 10 pens while distinguishing the two mixed pens from the others due to the unknown impact of the Lohmann LSL hens on egg-production.

Statistics

Welfare indicators

Statistical analyses were conducted in R version 3.6.1. To evaluate treatment effects on hen’s welfare indicators, we fitted one linear mixed-effect model from the ‘lme4’ package per welfare indicator (body mass, KBF severity the feather damage) as a function of date (or health assessment identity), treatment, and date-treatment interaction. We controlled for class and included hen identity nested in pen identity as a random term. Pen identity was removed when fitting the KBF severity and body mass due to low variance leading to convergence issues. We scaled body mass to be within 0-1 within each welfare assessment separately prior to the model fit. Model assumptions were checked visually (normality and homoscedasticity of residuals). To assess significance of the date-treatment interaction, we compared each model with a model that did not contain the interaction variable using the function Anova from R. When the date-treatment interaction was significant ($p < 0.05$), we reported results from a post-hoc analysis with adjusted p-values (Bonferroni adjustment, package “emmeans”). When the date-treatment interaction was not significant, we removed it from the model and assessed significance of the treatment as a main effect by comparing the full and reduced models.

Spatial behaviours

To evaluate whether treatment groups differed in mean behaviours, we fitted one generalized linear mixed-effect model from the package 'glmmTMB' [26] for each behaviour and month in the laying barn (10 months, six behaviours, total of 60 models), as a function of the treatment (with STAN as reference group). We chose months as the unit to analyse treatment effect over time, aiming to strike a balance between thoroughly estimating mean effects over time and avoiding potential noise linked to shorter time intervals like weeks. A previous study on the same hens, studying intra-individual variation in a composite behaviour, found that, on average, hens increased their indoor movements for 39 days after the transfer to the laying barn [22]. This previous result suggests that the transfer to the laying barn could have a long-term effect on hens' spatial behaviours. However, we expected that any treatment differences in spatial behaviours would appear directly after the transfer to the laying barn and diminish over time. Therefore, any statistically significant treatment effect that does not follow that pattern was interpreted with care, and the results section emphasized coefficient estimates rather than p-values. We controlled for the class, time (defined as the number of days since the transfer to the laying barn), KBF severity, body mass, and number of hours with artificial lights on, by including them as fixed effects. We interpolated linearly (with monotonically increasing) both the KBF severity score and body mass for each hen separately to better control for their health between two consecutive health assessments considering that both scores exhibit an upward trend over time. The hen identity nested in the pen identity was included as a random term. To avoid convergence issues due to the very low explained variance by the pen identity, we performed a likelihood ratio test with and without the pen identity and chose the full model when the p-value was < 0.05. For the WG-related behaviour, we also controlled for the number of hours the WG was accessible and the average daily external temperature (°C), taken from the LSZB weather station (~12 km from the barn) and accessed via the Wolfram alpha API in Python. All continuous variables were scaled by two times the deviation to obtain coefficients comparable to those of binary predictors (i.e., the treatment) [27]. As the first full day in the laying barn coincided with the final day of September, we incorporated that day into the models for the first month (October).

The vertical travelled distance was modelled with a gaussian family for months 2-10 and with a zero-inflated Poisson model with the rescaled number of days in the barn as the zero-inflation parameter for month 1. The nestbox tier usage was modelled with a gamma family (with a log-link function) and the WG presence with a binomial family (with a logit-link function). The proportion of indoor time spent on the top and nestbox tiers were both modelled with a *beta* family (with a logit-link function). The proportion of the indoor time spent on the littered floor was modelled with a gaussian family for months 2-10 and with a binomial family (with a logit-link function) to account for the excess of zeros (19.7% of observations) for month 1. Behaviours used in a beta distribution were first rescaled between 0.01 and 0.99. During the first month in the laying barn, hens had not reached the peak of production and the artificial light was turned on later in the day (days 1-9 at 9h,

days 10-16 at 8h, days 17-22 at 7h, days 23-24 at 6h, days 25-30 at 5h, days 31-32 at 4h). Therefore, we did not analyse the first month in the laying barn for the two nestbox tier related behaviours because behaviours expressed during that first month would likely not be comparable with behaviours expressed in subsequent months. In addition, we did not use the first seven days when fitting the WG presence, as the opening of the WG was delayed until normal laying behavior commenced (i.e., the eighth day) as is common for commercial practices. Residuals were simulated using the 'DHARMa' package to verify model assumptions (normality and homoscedasticity of the residuals). We reported the bootstrapped coefficients [28], credible intervals, and p-values computed from the 'parameters' package using 500 iterations.

Egg-production data

Because egg production is characterized at the pen-level (225 hens/pen) and because we have only five pens per treatment, we decided not to analyse the data statistically, as for instance by fitting the egg count per live hen as a function of treatment, age, and pen identity. We believe that such an analysis could overestimate the effect of treatment due to the lack of control for individual effects (e.g, the influence could come from only a few hens). Thus, we analysed the data for each pen separately and provided pen-level descriptive statistics. First, we analysed the data during the onset of lay, here defined as the first 60 days in the laying barn), an important period in which we might observe the greatest variation between hens. We fitted one sigmoid curve per pen: $\frac{a}{1+exp\left(\frac{m-time}{s}\right)}$, with *time* represented by the number of days since being in the laying barn, *a* as the horizontal asymptote, *m* as the time point at *a*/2, and *s* as the steepness of the curve at *a*/2 [29]. Sigmoid curves were fitted using a nonlinear least square algorithm (nls function in R). For each pen, we reported *a* as an indication of the level at which egg-production stabilises and *m* as an indication of the time point with the steepest slope, that is at the inflection point of the curve. Second, we provide for each pen, descriptive statistics including the distribution, the second and third quartiles and the median value of the average daily number of eggs per live hen, on the remaining period (days 61 in the laying barn until the end of production, that is 330 days after the transfer to the laying barn).

Results

Welfare indicators

The date-treatment interaction was neither a predictor of body mass ($x^2_{6,1121} = 9.49, p = 0.15$), feather damage ($x^2_{2,506} = 4.95, p = 0.08$), or KBF severity ($x^2_{3,669} = 4.34, p = 0.23$) and was therefore removed from all models. Treatment as a main effect was neither a predictor of body mass ($x^2_{1,1121} = 1.95, p = 0.16$) or feather damage ($x^2_{1,506} = 0.54, p = 0.46$), but was a predictor of KBF severity ($x^2_{1,669} = 6.35, p = 0.01$). Specifically, holding all else equal, the KBF severity score of OFH hens was, on average, 4.53 ([95% CI] = [0.99, 8.07]) points higher than that of STAN hens. All model estimates and p-values are presented in Table S1. The

observed scores of each welfare indicator per treatment and date are illustrated in a violin plot (Figure 1), and their means (±SD) presented in Table 1.

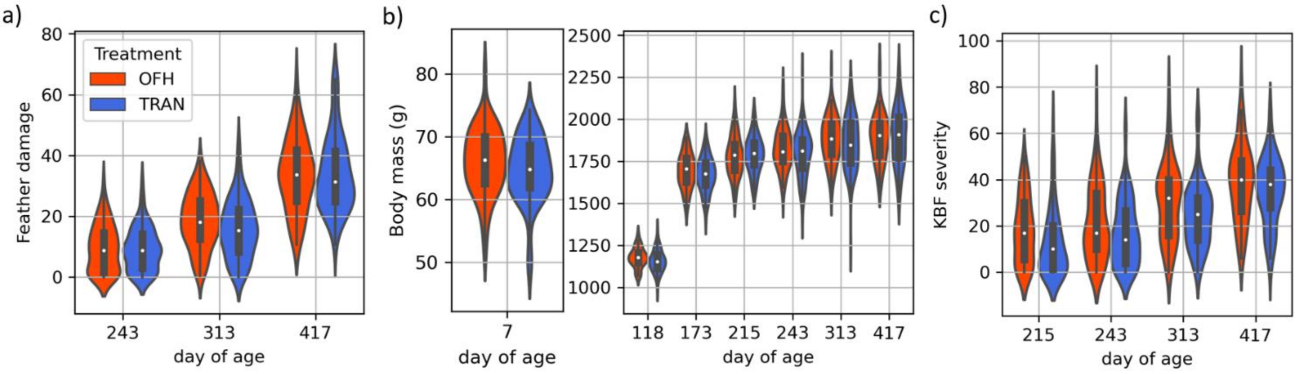

Figure 1 – Violin plot of the raw welfare indicators over day of age per treatment, including the feather damage (a), body mass (b), and KBF severity (c). Blue and red colours indicate the treatment groups, STAN and OFH, respectively.

	Feather damage		Body mass (g)		KBF severity	
Day of age	OFH	STAN	OFH	STAN	OFH	STAN
7			66.04 ±5.17	64.75 ±5.12		
118			1177.75 ±57.88	1157.16 ±64.90		
173			1692.88 ±103.13	1671.03 ±99.26		
215			1780.19 ±118.01	1790.72 ±105.09	18.35 ±14.16	12.72 ±14.57
243	9.58 ±7.73	9.24 ±7.02	1822.38 ±122.70	1795.32 ±137.46	21.50 ±16.21	16.86 ±13.98
313	18.57 ±8.92	15.83 ±9.59	1877.29 ±134.15	1855.84 ±160.46	30.20 ±16.26	24.57 ±13.68
417	33.60 ±11.79	34.09 ±12.31	1888.44 ±139.77	1898.60 ±168.97	38.64 ±16.34	34.94 ±14.25

Table 1 – Mean ±SD of the feather damage, body mass (in grams), and KBF severity for the OFH and STAN treatment groups.

Spatial behaviours

Models' coefficients from the treatment predictor for each behaviour across months are displayed in figure 2 (bootstrapped p-values and coefficients are detailed in supplementary Table S2). To help further interpreting the nature of the change, particularly in cases where a treatment effect was found for several months only (e.g., determining which, if any, of the treatment group had their behavioural responses *converging* toward those of the other group), the mean observed behavioural scores are presented per treatment and month in Table 2.

We can interpret exponentiated coefficients from our models with a beta family, that is, ones with the proportion of time spent on the top tier and the nestbox tier responses, as odds ratios. For example, during the first month in the laying barn, the odds of being on the top tier for OFH hens was 1.49 times that of STAN hens (95% CI [1.12, 2.04], $p = 0.01$; figure 2a), indicating that on average OFH hens spent more time on the top tier. A similar effect was found for month 2 (β [95% CI] = 1.67 [1.14, 2.35], $p < 0.01$). Similar interpretations

can be made for models with a proportion of time spent on the top tier or nestbox tier as response variables, although neither models related to the nestbox tier (figure 2c, 2f) nor to the top tier (figure 2a) had a significant effect of treatment beyond the second month.

We can interpret the exponentiated coefficients from our models with a binary response, that is, ones with the proportion of time spent on the littered floor (during month 1) and the WG presence responses, as odds ratios. For instance, during the first month in the laying barn, our results indicate that OFH hens were less likely to go to the littered floor compared to STAN hens. More specifically, we found an odds ratio of 0.30 (95% CI [0.12, 0.67], $p < 0.01$), meaning that the odds of going on the littered floor at least once during the day (during the first month) in OFH hens were 0.30 times the odds in STAN hens. A similar effect was maintained during months 2 and 3 (with a gaussian family; figure 2b). More specifically, compared to STAN hens, OFH hens spent 7% and 5% less of their daily indoor time on the littered floor during months 2 and 3, respectively (month 2: β [95% CI] = -0.07 [-0.11, -0.02], $p = 0.008$; month: 3 β [95% CI] = -0.05 [-0.11, -0.00], $p = 0.04$). Treatment was not a significant predictor of WG presence (see figure 2d).

Furthermore, we found that OFH hens moved less vertically during the first three months (see figure 2e). More specifically, hatching on farm was associated with a reduction of 31% in the vertical travelled distance during month 1 ($\exp(\beta)$ [95% CI] = 0.69 [0.59, 0.82], $p < 0.001$, Poisson distribution). The treatment effect persisted up to month 3, with OFH hens crossing on average 11 and 9 fewer zones per day than STAN hens during months 2 and 3, respectively (month 2: β [95% CI] = -10.62 [-16.13, -4.35], $p < 0.001$; month 3: β [95% CI] = -9.19 [-16.13, -2.50], $p = 0.012$, Gaussian distribution).

Lastly, OFH hens, on average, were slightly earlier in their nestbox tier timing during the second month than STAN hens (β [95% CI] = 0.96 [0.92, 0.99], $p < 0.05$; figure 2f). The treatment showed no effect in subsequent months in the nestbox tier timing, and we found no effect of treatment on the proportion of time spent on the nestbox-tier.

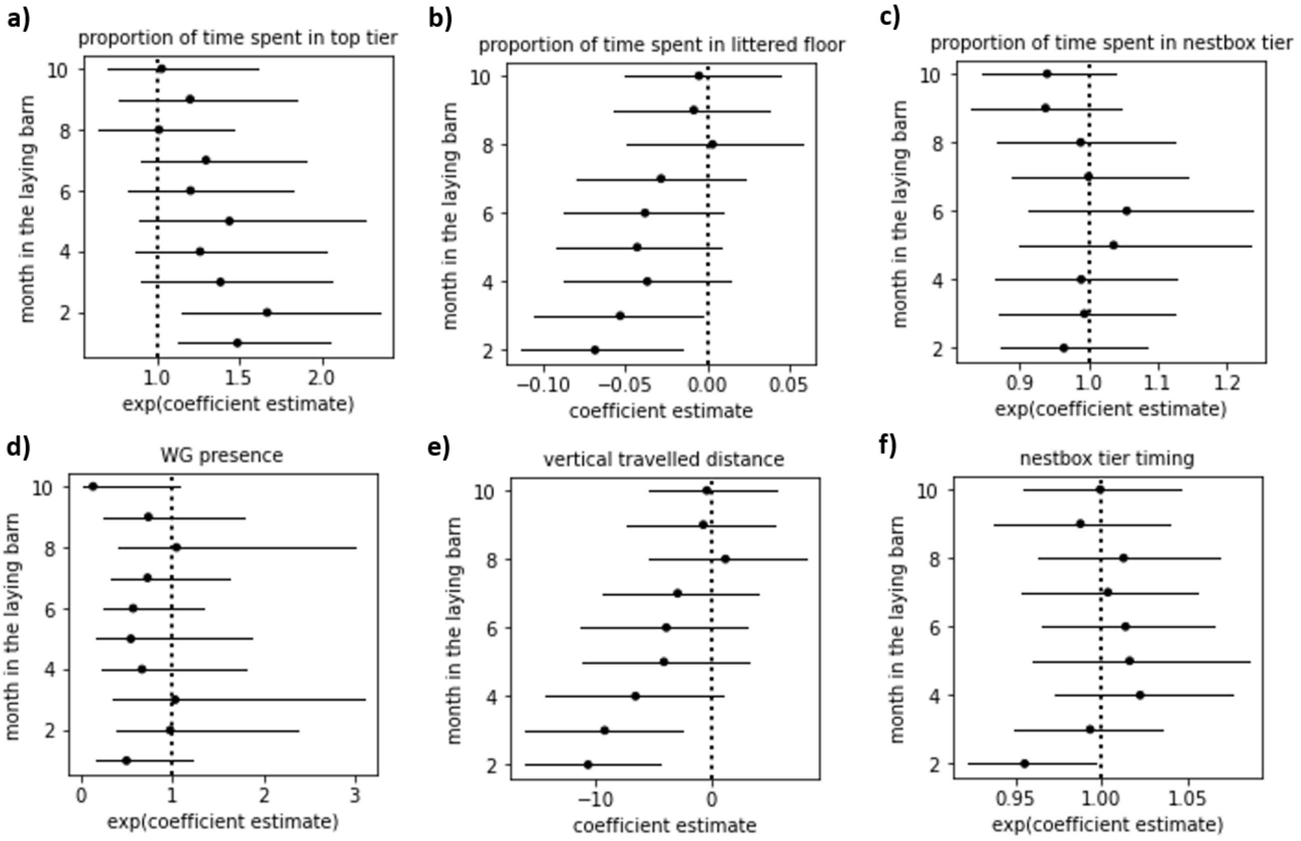

Figure 2 – Coefficient estimate of the treatment (with STAN as reference group) for each behaviour, for each of the months after the transfer to the laying barn (month 1-10). If the confidence interval includes the value of the dashed line, the treatment is not significant, else, if the point lies on the right side of the dashed line, it indicates that the model estimates higher behavioural response in OFH hens compared to STAN hens. Bootstrapped outputs (estimates and p-values) are detailed in supplementary Table S2.

month in laying barn	proportion of indoor time spent			WG presence	vert. travelled distance	nestbox tier timing
	top tier	littered floor	nestbox tier			
month1	**0.53 *vs.* 0.42**	**0.26 *vs.* 0.32**		0.44 *vs.* 0.51	**28.51 *vs.* 39.02**	
month2	**0.41 *vs.* 0.30**	**0.31 *vs.* 0.39**	0.07 *vs.* 0.07	0.62 *vs.* 0.65	**47.03 *vs.* 58.67**	**5.00 *vs.* 5.31**
month3	0.34 *vs.* 0.28	**0.36 *vs.* 0.41**	0.07 *vs.* 0.07	0.66 *vs.* 0.67	**55.43 *vs.* 63.99**	4.24 *vs.* 4.29
month4	0.33 *vs.* 0.27	0.38 *vs.* 0.42	0.08 *vs.* 0.08	0.62 *vs.* 0.69	61.64 *vs.* 68.68	4.19 *vs.* 4.14
month5	0.33 *vs.* 0.28	0.37 *vs.* 0.42	0.08 *vs.* 0.08	0.61 *vs.* 0.70	63.03 *vs.* 68.94	4.16 *vs.* 4.18
month6	0.30 *vs.* 0.24	0.40 *vs.* 0.44	0.09 *vs.* 0.09	0.65 *vs.* 0.73	66.50 *vs.* 72.47	4.46 *vs.* 4.52
month7	0.31 *vs.* 0.28	0.40 *vs.* 0.41	0.09 *vs.* 0.09	0.62 *vs.* 0.69	61.38 *vs.* 65.76	4.59 *vs.* 4.62
month8	0.30 *vs.* 0.29	0.41 *vs.* 0.41	0.08 *vs.* 0.08	0.69 *vs.* 0.72	58.36 *vs.* 58.61	4.78 *vs.* 4.74
month9	0.28 *vs.* 0.26	0.43 *vs.* 0.43	0.08 *vs.* 0.09	0.71 *vs.* 0.83	57.11 *vs.* 58.38	5.05 *vs.* 5.18
month10	0.27 *vs.* 0.26	0.43 *vs.* 0.43	0.08 *vs.* 0.08	0.68 *vs.* 0.80	52.95 *vs.* 53.14	5.34 *vs.* 5.36

Table 2 – Mean observed behaviours exhibited by the OFH *vs.* STAN hens for the six spatial behaviours (proportion of indoor time spent on the top tier, littered floor, and nestbox tier, the WG presence, the vertical travelled distance, and the nestbox tier timing). We highlighted in bold the months and behaviours for which we found a treatment effect.

Egg-production data

The sigmoid curve of the daily average number of eggs per live hen in each pen during the first 60 days in the laying barn and their associated parameters *a* and *m* are illustrated in Figure 3a and 3b, respectively. The level at which the average number of daily eggs per live hen stabilised during the first 60 days in the laying barn (as measured by the *a* estimate) for OFH pens was between 0.948 and 0.964, and slightly lower for STAN pens (0.926 - 0.956). The time point (i.e., number of days since transfer to the laying barn) at the inflection point of the curve (as measured by the *m* estimate) is between 24.05 and 25.79 for OFH pens and slightly earlier for STAN pens between 23.71 and 25.00. The average daily number of eggs per hen in the laying barn after the first 60 days in the laying barn until the end of production is illustrated in Figure 3c and 3d for STAN and OFH hens, respectively. Values above one may occur when some eggs are collected the day after being laid.

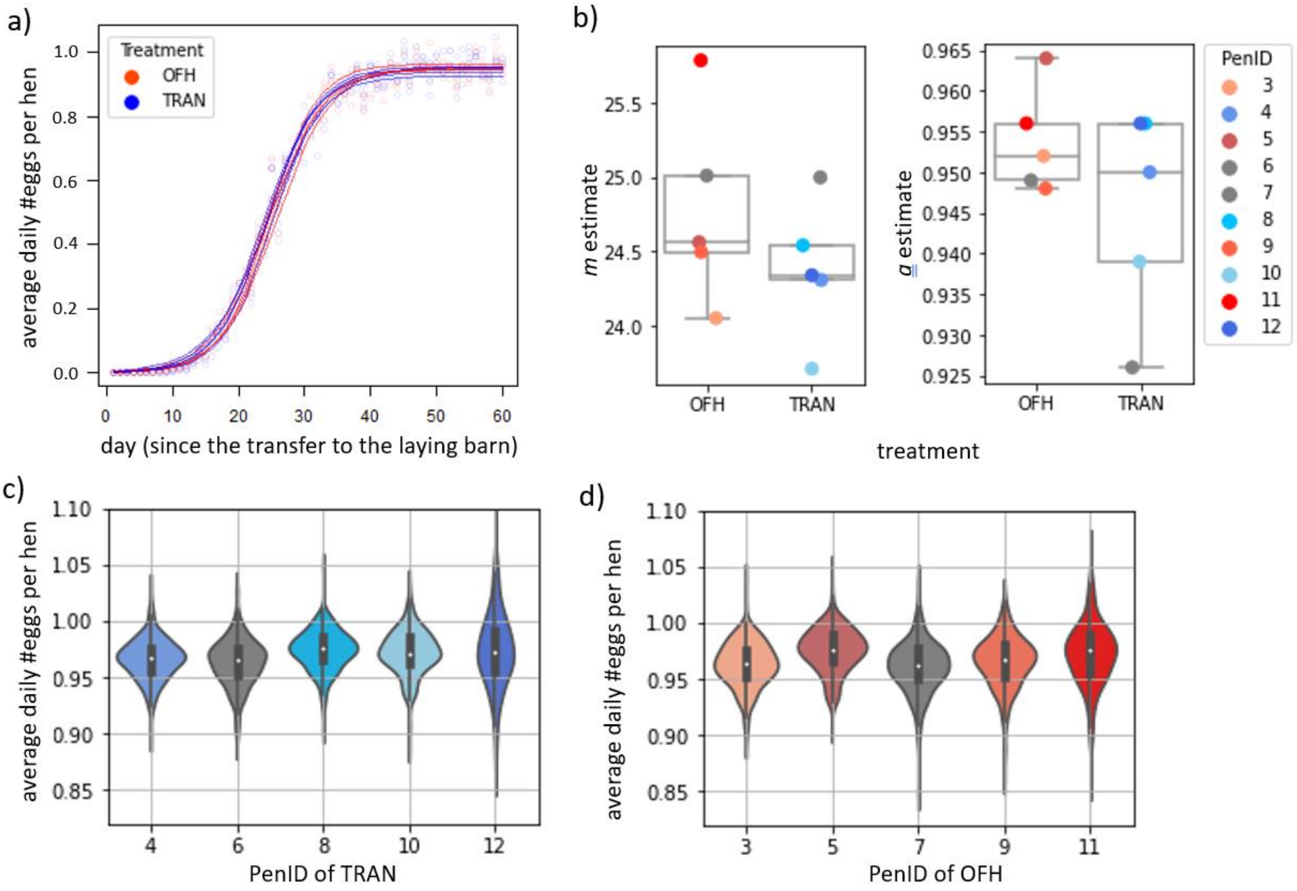

Figure 3 – Average daily number of eggs per hen, with the associated growth curve (for each pen) during the first 60 days in the laying barn (a), their associated parameters *a* (as an indication of the level at which egg production stabilise) and *m* (as an indication of the time point at the inflection point of the curve) (b), and the raw data per pen from the second month in the laying barn until the end of production for STAN hens (c) and OFH hens (d). Blue and red colours are indicative of STAN and OFH hens, respectively. The two special pens containing 205 animals of the one treatment group but also an additional 20 Lohmann LSL hens, are highlighted in grey.

Discussion

In this study, we found that on average, OFH hens had more severe KBF throughout the laying period, transitioned less between the aviary tiers and spent less time on the littered floor and more on the top tier compared to STAN hens. We found no treatment effect on the daily presence in the WG, almost none on the two nestbox tier related behaviours, and none in body mass or feather damage. To our knowledge, this study is the first to investigate the potential effects of the standard commercial hatchery process throughout the laying period on commercial hens' welfare and spatial behaviour, and in particular to assess the effect on KBF. These findings suggest that early-life stressors associated with the commercial hatchery environment – here, transport-related stressors and temporary post-hatch resource deprivation – may have prepared hens for their adult environment and thereby support the environmental matching hypothesis.

Welfare indicators

According to the environmental matching hypothesis, early-life stress can prepare an individual for similar adversities encountered in adulthood [13]. The adulthood of commercial laying hens is typically accompanied by aversive situations, including the transfer to the laying barn. The potential stressors associated with this transfer, such as being transported to a new housing with new conspecifics, echo those encountered by STAN chicks. Therefore, the adult environment of commercial hens may better match with the environmental conditions experienced by day-old STAN chicks rather than those of day-old OFH chicks.

The result showing less severe KBF in STAN hens across the laying period compared to OFH hens provides support for the environmental matching hypothesis. Although KBF are considered as one of the greatest welfare issues in the egg production industry [17,18,30–34], effects of early-life stressors induced by commercial hatchery on KBF was to our knowledge not yet investigated. Various factors are thought to contribute to the prevalence of KBF, including genetic predisposition [35], nutrition [35], de-mineralized bone aggravated by the high egg laying rate [36–38], inactivity compromising bone health [39], and certain physical elements of the housing system that may cause pressure on the bone when hens are perching [40] or lead to trauma resulting from impact collisions [21,39,41,42]. Given that the STAN and OFH hens originated from the same parent flock, it is unlikely that genetic predisposition could explain this result. Moreover, apart from the nutritional differences after hatching, where OFH chicks had immediate access to feed and water, which is unlikely to negatively impacted their bone health, both STAN and OFH hens received the same nutrition. Because we observed minor differences in egg-production during the onset of lay, which, if anything, would suggest that OFH hens had a slower onset of lay, it is also unlikely that these differences would explain the result.

Given the limited amount of genetic and environmental differences between treatment groups outside of the treatment specific differences, we believe behavioural differences in activity and/or different

usage of the housing system could explain why OFH hens had more severe KBF compared to STAN hens. For instance, our finding indicating a 31% reduction in vertical travelled distance during the first month for hens hatched on farms, may reflect a more general pattern of inactivity among OFH hens which could lead to poorer bone health and in turn higher KBF [39]. Additionally, OFH hens spent more time on the top tier, where hard metal perches are more abundant. High perch use would result in overall greater pressure on the keel bone leading to deviated keels, which in turn can weaken the keel's overall structural integrity [41]. Overall, we cannot exclude the possibility that the treatment difference we observed in the severity of KBF may be an indirect effect of the differences we observed in spatial behaviours between the treatment groups. Further research is required to replicate this finding and to understand the underlying mechanisms involved.

We found that OFH hens had on average a similar body mass to STAN hens beginning with our earliest measurement at 7 DOA. We expected OFH hens to weigh more because of direct access to feed after hatching, which previous studies on day-old chicks demonstrated [3,9,10], but also because of the potential decreased weight gained due to early life stress [43,44]. However, beyond one day of age, the effect in previous literature is ambiguous. Studies contrasting on-farm hatching to standard commercial practices found no treatment differences at 4 or 7 DOA [3,9], a tendency for on-farm hatched chicks to weigh more up to 11 weeks of age [10], and that on-farm hatched chicks weighed less at 8, 15, 22 and 29 DOA [2]. Our study provides more evidence that treatment effects of body mass are not present during lay.

Furthermore, OFH hens had similar feather damage to STAN hens, which supports neither the silver-spoon nor the environmental matching hypothesis, but is in line with the inconclusive findings in earlier literature that reported both positive [2] and negative [9] effects. The ambiguity surrounding these results may stem from the multifactorial nature of feather damage, which includes factors such as feather pecking and abrasion resulting from different parts of the structures [45]. Therefore, the influences of the physical and social environments on feather damage could outweigh or interact with effects from early-life stressors.

Spatial behaviours

The literature on behavioural differences between hens that hatched on-farm versus in the standard commercial hatchery is sparse and mainly conducted in test arenas [2,3,46–48], hindering the extrapolation of the results to commercial settings, typically characterized by more complex housing systems and larger groups. In this study, we used tracking technology to monitor movements of hens within a quasi-commercial aviary system throughout the laying period.

We found that for the first three months post-transfer to the laying barn (up to 7-month-old) STAN hens spent more time on the littered floor and less on the top tier. The top tier has been shown to be used more extensively by hens with more severe keel bone fractures [49] and throughout full days following their transfer to the laying barn [22]. Thus, it is possible that hens use this area over the day to offset stress or pain.

Unlike the top tier, the littered floor promotes a diverse range of natural behaviours, including locomotion, dust bathing, exploring, foraging, and scratching. Therefore, these findings could indicate that OFH hens exhibited fewer natural behaviours compared to STAN hens in this early period. Furthermore, as OFH hens became behaviourally more similar to STAN hens over time (Table 2), these results further suggest that OFH hens required a prolonged period to establish their daily behavioural patterns. Overall, STAN hens may have exhibited better abilities in coping with the transfer to the laying barn, which would provide further support for the environmental matching hypothesis. Early-life stress can enhance behavioural flexibility [50] and facilitate spatial learning and memory [50,51], especially when experienced close in time and within the same context that is encountered later [52]. Therefore, increased stress induced by the commercial hatchery process near the time of transfer to the rearing barn could have induced focused attention and improved the memory of relevant information allowing STAN hens to better cope to the laying barn.

In addition to the potential effect of early-life stressors, it is possible that the STAN chicks may have exhibited faster acclimatization to their new environment due to the inherent effects of their transition from the hatchery to the farm. As suggested by Skånberg et al. [53], it is possible that environmental change during rearing plays a role in enhancing layer chicks' adaptability later in life. Specifically, STAN hens may have learned appropriate cognitive and locomotive skills during the rearing period, facilitating their adaptation to the aviary system. However, a previous study comparing the cognitive ability of layer chicks that had temporary post-hatch resource deprivation and eight-hour transport, to those that had ad libitum access to feed and water and were not transported, found no treatment effect in a cognitive test [10]. Further research is needed to investigate whether STAN hens possess better spatial-cognitive abilities in the laying period.

It is also possible that this increased early-life stress has altered the functional and structural development of the Hypothalamic-Pituitary-Adrenal (HPA)-axis [54,55]. Typically, stress induced in the early postnatal period results in HPA-axis hyper-reactivity during adulthood [54–57], with enhanced depression-like behaviours and anxiety [58]. Previous studies on laying hens generally supports such alterations of the HPA-axis. It was shown that hens from a commercial hatchery had a more pessimistic cognitive judgement bias, a more sensitive HPA-axis, and a stronger reaction to stressors during the first weeks of life compared to on-farm hatched hens [2,47]. Therefore, we would anticipate STAN hens to exhibit stronger stress responses and potentially adopt a more pessimistic perception of the situation compared to OFH hens. However, since we found that STAN hens spent more time on the littered floor and less on the top tier and displayed their typical behavioural patterns earlier on relatively to OFH hens, it is probable that the early-life stress did not affect the HPA-axis of STAN hens. Instead, we propose that later potential stressors experienced by hens from both treatment groups in the rearing barn would have counterbalanced potential alterations of the HPA-axis [54]. However, future research comparing indicators of stress levels between OFH and STAN hens during both the rearing and laying periods is necessary to substantiate our interpretation.

We found almost no treatment effect on the two nestbox tier behaviours, which supports the idea of limited behavioural plasticity related to the use of the nestbox tier. Given the strong human selection for high productivity and the high motivation to use nestboxes [59], it is possible that those behaviours are more tightly correlated with physiology or with strong animal needs and thus are less plastic than others. That is, these behaviours may be less influenced by external or internal factors and more repeatable across different contexts, as suggested by previous findings [49,60].

Egg-production data

Because of the expected increase in on-farm hatching practices due to the development of *in ovo* sexing technique [61], there is a need for studies comparing egg-production between OFH and STAN hens. To our knowledge, only two studies [2,9] have undertaken this task, by collecting daily production data at the pen-level. The authors found that OFH hens had a slower onset of egg laying than STAN hens at 15-20 weeks of age [2] in one study and laid more and bigger eggs at 19-25 weeks of age in the second [9]. Due to the limited amount of data, we were unable to test the treatment's effect on egg production statistically, but our results at 17-25 weeks of age could suggest that OFH hens have a slower onset of lay with compensation of more eggs per live hen later on. These observations support the findings of the two previous studies [2,9], however, further research comparing egg-production between OFH and STAN hens with substantially more pens or at the individual-level is required to confirm these observations.

Conclusion

Hens from both treatments originated from the same parent flock yet produced different phenotypes depending on their early-life environments, suggesting the presence of developmental plasticity in our commercial hens. By providing nuance to the relative benefit of on-farm hatching for animal welfare described by previous literature, this study highlighted the critical role in designing early-life environments in accordance with the adult environment. For laying hens housed in aviary systems, our results could suggest that hatching on-farm rather than in commercial hatchery may lead to an increased mismatch between the hatching and laying environments that can be detrimental to animal welfare. We conclude that future research is needed to determine whether conventional husbandry practices, originally designed for hens hatched in commercial hatcheries, need to be adapted for hens hatched on-farm, such as implementing smoother transfers between rearing to laying barns.

Acknowledgements

We are thankful to Abdulsatar Abdel Rahman for his daily checks on the tracking system and the Aviforum staff for animal care and egg-production data.

References

[1] FAO. Food and Agricultural Organization of the United Nations n.d. http://www.fao.org (accessed January 4, 2023).

[2] Hedlund L, Whittle R, Jensen P. Effects of commercial hatchery processing on short- and long-term stress responses in laying hens. Sci Rep 2019;9:1–10. https://doi.org/10.1038/s41598-019-38817-y.

[3] Hedlund L, Jensen P. Incubation and hatching conditions of laying hen chicks explain a large part of the stress effects from commercial large-scale hatcheries. Poult Sci 2021;100:1–8. https://doi.org/10.1016/J.PSJ.2020.10.015.

[4] Grafen A. On the uses of data on lifetime reproductive success. In: T. H. Clutton-Brock, editor. Reproductive success, vol. 363, 1988, p. 454–71.

[5] Cooper EB, Kruuk LEB. Ageing with a silver-spoon: A meta-analysis of the effect of developmental environment on senescence. Evol Lett 2018;2:460–71. https://doi.org/10.1002/EVL3.79.

[6] De Jong IC, van Hattum T, van Riel JW, De Baere K, Kempen I, Cardinaels S, et al. Effects of on-farm and traditional hatching on welfare, health, and performance of broiler chickens. Poult Sci 2020;99:4662–71. https://doi.org/10.1016/J.PSJ.2020.06.052.

[7] De Jong IC, Gunnink H, Van Hattum T, Van Riel JW, Raaijmakers MMP, Zoet ES, et al. Comparison of performance, health and welfare aspects between commercially housed hatchery-hatched and on-farm hatched broiler flocks. Animal 2019;13:1269–77. https://doi.org/10.1017/S1751731118002872.

[8] Giersberg MF, Molenaar R, de Jong IC, Souza da Silva C, van den Brand H, Kemp B, et al. Effects of hatching system on the welfare of broiler chickens in early and later life. Poult Sci 2021;100:100946. https://doi.org/10.1016/J.PSJ.2020.12.043.

[9] Hedlund L, Jensen P. Effects of stress during commercial hatching on growth, egg production and feather pecking in laying hens. PLoS One 2022;17. https://doi.org/10.1371/JOURNAL.PONE.0262307.

[10] Witjes VL, Bruckmaier RM, Gebhardt-Henrich SG, Toscano MJ. Effects of on-farm hatching on short term stress indicators, weight gain, and cognitive ability in layer chicks. Appl Anim Behav Sci 2022:105692. https://doi.org/10.1016/J.APPLANIM.2022.105692.

[11] Lea AJ, Tung J, Archie EA, Alberts SC. Developmental plasticity: Bridging research in evolution and human health. Evol Med Public Health 2017;2017:162. https://doi.org/10.1093/EMPH/EOX019.

[12] Gluckman PD, Hanson MA, Spencer HG, Bateson P. Environmental influences during development and their later consequences for health and disease: implications for the interpretation of empirical studies.

Proceedings of the Royal Society B: Biological Sciences 2005;272:671–7. https://doi.org/10.1098/RSPB.2004.3001.

[13] Monaghan P. Early growth conditions, phenotypic development and environmental change. Philosophical Transactions of the Royal Society B: Biological Sciences 2007;363:1635–45. https://doi.org/10.1098/RSTB.2007.0011.

[14] Pigeon G, Loe LE, Bischof R, Bonenfant C, Forchhammer M, Irvine RJ, et al. Silver spoon effects are constrained under extreme adult environmental conditions. Ecology 2019;100:e02886. https://doi.org/10.1002/ECY.2886.

[15] Giersberg MF, Spindler B, Kemper N. Assessment of Plumage and Integument Condition in Dual-Purpose Breeds and Conventional Layers. Animals 2017, Vol 7, Page 97 2017;7:97. https://doi.org/10.3390/ANI7120097.

[16] Drake KA, Donnelly ProfCA, Dawkins MS. Influence of rearing and lay risk factors on propensity for feather damage in laying hens. Br Poult Sci 2010;51:725–33. https://doi.org/10.1080/00071668.2010.528751.

[17] Baur S, Rufener C, Toscano MJ, Geissbühler U. Radiographic Evaluation of Keel Bone Damage in Laying Hens—Morphologic and Temporal Observations in a Longitudinal Study. Front Vet Sci 2020;0:129. https://doi.org/10.3389/FVETS.2020.00129.

[18] Rufener C, Makagon MM. Keel bone fractures in laying hens: a systematic review of prevalence across age, housing systems, and strains. J Anim Sci 2020;98:S36–51. https://doi.org/10.1093/JAS/SKAA145.

[19] Gunnarsson S, Matthews LR, Foster TM, Temple W. The demand for straw and feathers as litter substrates by laying hens. Appl Anim Behav Sci 2000;65:321–30. https://doi.org/10.1016/S0168-1591(99)00065-9.

[20] Hemsworth PH, Edwards LE, Hemsworth PH, Edwards LE. Natural behaviours, their drivers and their implications for laying hen welfare. Anim Prod Sci 2020;61:915–30. https://doi.org/10.1071/AN19630.

[21] Stratmann A, Fröhlich EKF, Gebhardt-Henrich SG, Harlander-Matauschek A, Würbel H, Toscano MJ. Modification of aviary design reduces incidence of falls, collisions and keel bone damage in laying hens. Appl Anim Behav Sci 2015;165:112–23. https://doi.org/10.1016/j.applanim.2015.01.012.

[22] Montalcini CM, Toscano MJ, Gebhardt-Henrich SG, Petelle MB. Intra-individual variation of hen movements is associated with later keel bone fractures in a quasi-commercial aviary. Sci Rep 2023;13:2377. https://doi.org/10.1038/S41598-023-29587-9.

[23] Tauson R, Kjaer J, Maria GA, Cepero R, Holm K-E. The creation of a common scoring system for the integument and health of laying hens: Applied scoring of integument and health in laying hens. Final report Health from the Laywell project 2005. https://www.laywel.eu/web/pdf/deliverables%2031-33%20health.pdf (accessed December 22, 2022).

[24] Rufener C, Baur S, Stratmann A, Toscano MJ. A Reliable Method to Assess Keel Bone Fractures in Laying Hens From Radiographs Using a Tagged Visual Analogue Scale. Front Vet Sci 2018;5:124. https://doi.org/10.3389/fvets.2018.00124.

[25] Montalcini CM, Voelkl B, Gómez Y, Gantner M, Toscano MJ. Evaluation of an Active LF Tracking System and Data Processing Methods for Livestock Precision Farming in the Poultry Sector. Sensors 2022;22:659. https://doi.org/10.3390/S22020659.

[26] Brooks ME;, Kristensen K;, Van Benthem KJ;, Magnusson A;, Berg CW;, Nielsen A;, et al. glmmTMB balances speed and flexibility among packages for zero-inflated generalized linear mixed modeling. R J 2017;9:378–400. https://doi.org/10.3929/ETHZ-B-000240890.

[27] Gelman A. Scaling regression inputs by dividing by two standard deviations. Stat Med 2008;27:2865–73. https://doi.org/10.1002/SIM.3107.

[28] Davison A, Hinkley D. Bootstrap methods and their application. Cambridge University Press; 1997.

[29] Caroli A, Frisoni GB, Initiative TADN. The dynamics of Alzheimer's disease biomarkers in the Alzheimer's Disease Neuroimaging Initiative cohort. Neurobiol Aging 2010;31:1263. https://doi.org/10.1016/J.NEUROBIOLAGING.2010.04.024.

[30] Rodenburg T, Tuyttens F, de Reu K, Herman L, Zoons J, Sonck B. Welfare assessment of laying hens in furnished cages and non-cage systems: an on-farm comparison. Animal Welfare 2008;17:363–73.

[31] Armstrong EA, Rufener C, Toscano MJ, Eastham JE, Guy JH, Sandilands V, et al. Keel bone fractures induce a depressive-like state in laying hens. Sci Rep 2020;10:1–14. https://doi.org/10.1038/s41598-020-59940-1.

[32] Nasr MAF, Nicol CJ, Murrell JC. Do Laying Hens with Keel Bone Fractures Experience Pain? PLoS One 2012;7:e42420. https://doi.org/10.1371/JOURNAL.PONE.0042420.

[33] Harlander-Matauschek A, Rodenburg TB, Sandilands V, Tobalske BW, Toscano MJ. Causes of keel bone damage and their solutions in laying hens. Worlds Poult Sci J 2015;71:461–72. https://doi.org/10.1017/S0043933915002135.

[34] Riber AB, Casey-Trott TM, Herskin MS. The Influence of Keel Bone Damage on Welfare of Laying Hens. Front Vet Sci 2018;0:6. https://doi.org/10.3389/FVETS.2018.00006.

[35] Fleming RH, Mccormack HA, Mcteir L, Whitehead CC. Relationships between genetic, environmental and nutritional factors influencing osteoporosis in laying hens. Br Poult Sci 2007;47:742–55. https://doi.org/10.1080/00071660601077949.

[36] Randall CJ, Duff SR. Avulsion of the patellar ligament in osteopenic laying fowl. Vet Rec 1988;123:439–41. https://doi.org/10.1136/VR.123.17.439.

[37] Whitehead CC, Fleming RH. Osteoporosis in Cage Layers. Poult Sci 2000;79:1033–41. https://doi.org/10.1093/PS/79.7.1033.

[38] Fleming RH, McCormack HA, McTeir L, Whitehead CC. Incidence, pathology and prevention of keel bone deformities in the laying hen. Br Poult Sci 2004;45:320–30. https://doi.org/10.1080/00071660410001730815.

[39] Toscano MJ, Dunn IC, Christensen JP, Petow S, Kittelsen K, Ulrich R. Explanations for keel bone fractures in laying hens: are there explanations in addition to elevated egg production? Poult Sci 2020;99:4183–94. https://doi.org/10.1016/J.PSJ.2020.05.035.

[40] Pickel T, Schrader L, Scholz B. Pressure load on keel bone and foot pads in perching laying hens in relation to perch design. Poult Sci 2011;90:715–24. https://doi.org/10.3382/PS.2010-01025.

[41] Stratmann A, Fröhlich EKF, Harlander-Matauschek A, Schrader L, Toscano MJ, Würbel H, et al. Soft Perches in an Aviary System Reduce Incidence of Keel Bone Damage in Laying Hens. PLoS One 2015;10:e0122568. https://doi.org/10.1371/JOURNAL.PONE.0122568.

[42] Sandilands V, Moinard C, Sparks NHC. Providing laying hens with perches: fulfilling behavioural needs but causing injury? Br Poult Sci 2009;50:395–406. https://doi.org/10.1080/00071660903110844.

[43] Yahav S, Hurwitz S. Induction of Thermotolerance in Male Broiler Chickens by Temperature Conditioning at an Early Age. Poult Sci 1996;75:402–6. https://doi.org/10.3382/PS.0750402.

[44] Scanes CG. Biology of stress in poultry with emphasis on glucocorticoids and the heterophil to lymphocyte ratio. Poult Sci 2016;95:2208–15. https://doi.org/10.3382/PS/PEW137.

[45] Heerkens JLT, Delezie E, Kempen I, Zoons J, Ampe B, Rodenburg TB, et al. Specific characteristics of the aviary housing system affect plumage condition, mortality and production in laying hens. Poult Sci 2015;94:2008–17. https://doi.org/10.3382/PS/PEV187.

[46] Gabrielle L, Rebecca O, Louise H, Johanna G, Per J. Play ontogeny in young chickens is affected by domestication and early stress. Sci Rep 2022;12:1–8. https://doi.org/10.1038/s41598-022-17617-x.

[47] Hedlund L, Palazon T, Jensen P. Stress during commercial hatchery processing induces long-time negative cognitive judgement bias in chickens. Animals 2021;11:1083. https://doi.org/10.3390/ANI11041083/S1.

[48] Giersberg MF, Molenaar R, de Jong IC, De Baere K, Kemp B, Brand H van den, et al. Group level and individual activity of broiler chickens hatched in 3 different systems. Poult Sci 2023:102706. https://doi.org/10.1016/J.PSJ.2023.102706.

[49] Rufener C, Abreu Y, Asher L, Berezowski JA, Maximiano Sousa F, Stratmann A, et al. Keel bone fractures are associated with individual mobility of laying hens in an aviary system. Appl Anim Behav Sci 2019;217:48–56. https://doi.org/10.1016/j.applanim.2019.05.007.

[50] Calandreau L, Bertin A, Boissy A, Arnould C, Constantin P, Desmedt A, et al. Effect of one week of stress on emotional reactivity and learning and memory performances in Japanese quail. Behavioural Brain Research 2011;217:104–10. https://doi.org/10.1016/J.BBR.2010.10.004.

[51] Oitzl MS, Reichardt HM, Joëls M, De Kloet ER. Point mutation in the mouse glucocorticoid receptor preventing DNA binding impairs spatial memory. Proceedings of the National Academy of Sciences 2001;98:12790–5. https://doi.org/10.1073/PNAS.231313998.

[52] Joëls M, Pu Z, Wiegert O, Oitzl MS, Krugers HJ. Learning under stress: how does it work? Trends Cogn Sci 2006;10:152–8. https://doi.org/10.1016/J.TICS.2006.02.002.

[53] Skånberg L, Newberry RC, Estevez I, Keeling LJ. Environmental change or choice during early rearing improves behavioural adaptability in laying hen chicks. Sci Rep 2023;13:1–13. https://doi.org/10.1038/s41598-023-33212-0.

[54] Van Bodegom M, Homberg JR, Henckens MJAG. Modulation of the hypothalamic-pituitary-adrenal axis by early life stress exposure. Front Cell Neurosci 2017;11:87. https://doi.org/10.3389/FNCEL.2017.00087/BIBTEX.

[55] Stoneham ET, McHail DG, Samipour-Biel S, Liehr N, Lee CM, Evans JC, et al. Spatial Learning Is Impaired in Male Pubertal Rats Following Neonatal Daily but Not Randomly Spaced Maternal Deprivation. Front Cell Dev Biol 2021;9. https://doi.org/10.3389/FCELL.2021.621308.

[56] De Kloet ER, Joëls M, Holsboer F. Stress and the brain: from adaptation to disease. Nature Reviews Neuroscience 2005 6:6 2005;6:463–75. https://doi.org/10.1038/nrn1683.

[57] Ladd CO, Huot RL, Thrivikraman K V., Nemeroff CB, Plotsky PM. Long-term adaptations in glucocorticoid receptor and mineralocorticoid receptor mrna and negative feedback on the hypothalamo-pituitary-adrenal axis following neonatal maternal separation. Biol Psychiatry 2004;55:367–75. https://doi.org/10.1016/J.BIOPSYCH.2003.10.007.

[58] Maniam J, Antoniadis C, Morris MJ. Early-life stress, HPA axis adaptation, and mechanisms contributing to later health outcomes. Front Endocrinol (Lausanne) 2014;5:73. https://doi.org/10.3389/FENDO.2014.00073/BIBTEX.

[59] Cooper JJ, Albentosa MJ. Behavioural priorities of laying hens. Avian and Poultry Biology Reviews 2003;14:127–49.

[60] Montalcini CM, Petelle MB, Toscano MJ. Commercial laying hens exhibit long-term consistent individual differences and behavioural syndromes in spatial traits. R Soc Open Sci 2023;10. https://doi.org/10.1098/RSOS.230043.

[61] Preuße G, Porstmann V, Bartels T, Schnabel C, Galli R, Koch E, et al. Highly sensitive and quick in ovo sexing of domestic chicken eggs by two-wavelength fluorescence spectroscopy. Anal Bioanal Chem 2022:1–11. https://doi.org/10.1007/S00216-022-04446-0/TABLES/4.

Data Accessibility

The data and code for this study are available on https://doi.org/10.17605/OSF.IO/5WQGD.

Click here to access/download
Supporting Information
Supporting Information.docx
