# Supplementary material for: Commercial hatchery practices have long-lasting effects on laying hens’ spatial behaviour and health
Source: PLoS One. 2023 Dec 20;18(12):e0295560. doi: 10.1371/journal.pone.0295560 (PMC10732460; doi:10.1371/journal.pone.0295560)
Supplement: Attachment — Submitted filename: Response to Reviewers.docx [file pone.0295560.s009.docx]

Dear Prof. Lamiaa Mostafa Radwan and Reviewers,

Thank you very much for giving us the opportunity to revise our manuscript. We also thank the three reviewers for their kind comments and constructive suggestions. We believe their recommendations have significantly enhanced the statistical analysis, particularly in the production sub-section. It now includes a survival analysis based on the daily mortality and a statistical evaluation of the treatment's effect on egg production data. Thanks to reviewers’ suggestions, we also clarified different aspects of the Material and Methods, including the study design and the statistical analysis. The discussion now contains more detailed interpretations and further alternatives explanations (including how factors during egg-laying may relate to our results on keel bone fractures). We provide point-by-point responses below and hope the reviewers’ concerns have been addressed.

With best regards,

Camille Montalcini

## Editor Comments

1- Material and methods need more details, especially the method used to determine the sex of the newly hatched chicks.

*We now have in added more details in the study design, including the method used to determine the sex of the newly hatched chicks: L. 91-95:*

*“**Similar to OFH chicks, STAN chicks were vaccinated and manually sexed by examining their wing feathers for sex-specific patterns (this was however conducted at the hatchery facility). Although both OFH and TRAN chicks were manually sexed by the same company managing the post-hatch procedures (Prodavi SA, CH), we supervised the sorting of OFH chicks and encouraged the sexers to proceed gently, however no objective comparison was made of the handling procedures between treatment groups.”*

*We, for instance, also added more detailed on the hatching procedure on L.80-84:*

*“We monitored environmental conditions and temperature of 30 eggs every six hours throughout the hatching process. Specifically, we ensured that the ambient relative humidity remained above 30%, the windspeed below 0.15 m/s, ambient temperature above 32°C, and the eggshells temperature between 35-38 °C (see supplementary S1 Fig for eggshell temperature over time). Our methodology is similar to a previous study conducted on layer chicks* [1]*.”*

2- The statistical model used needs to be reviewed and written with more clarification and detail, and all data in the manuscript must be subjected to statistical analysis.

*Thank you, we agree that it was beneficial to clarify some aspects in the statistical section and to have added statistics for the egg-production data. We also included the daily mortality rate as suggested by reviewer3 and analysed it using survival analysis. We believe the egg-production subsection, which we have now renamed “production traits”, is greatly improved, and so is the manuscript.*

## ****Reviewer 1****

A very interesting paper on an important topic. I would like the authors to attend to the following comments and suggestions:

*Many thanks for the general positive appreciation and for your helpful suggestions across diverse themes, including KBF, statistical analysis of the egg-production, and the hatching-related procedures performed on the OFH chicks. We hope to have clarified each of your comments in the below point-by-point responses.*

- The eggs were transported on day 18 of incubation to the on-farm hatch system. Please consider the possible stress this may have induced on the embryos. Recent research has showed that the embryos may be susceptible to stress at this point and this may have affected your results (Nordquist, R.E., Vernooij, J.C.M., Dull, C.L., Pascual, A., van der Linde, G., Goerlich, V.C., 2022. The effects of transport of 18-day old hatching eggs on physiology and behaviour of slow growing broiler chicken. Applied Animal Behaviour Science 257.)

*Thank you very much for pointing this out to us and providing a reference. We have now added a paragraph in the discussion in a new sub-section named “****Limitation****”, because we believe that several aspects that you mentioned throughout your review related to the same idea, i.e., the limitations of the study. We believe these are all important aspects of the study that should be clearly mentioned. L. 467-479:*

*“This study aimed to assess the potential impact of commercial hatchery practices and potential benefits of on-farm hatching for animal welfare within commercially relevant settings throughout most of the production period. Some potential early life stressors were in this study uniquely experienced by STAN animals, including food and water deprivation* [2] *and the transportation at one day of age to a new environment* [3]*. Yet, the hatchery-related procedures applied to OFH chicks might also have been aversive, including the transportation at 18 days of incubation* [4] *and the vaccination and sexing at one day of age.* *We designed the experiment so that all hatchery-related procedures should be less, or when not possible, equally, aversive for the OFH treatment compared to the TRAN treatment. However, our methodology did not allow to determine if the increased cumulative adversities encountered by STAN chicks during their development led to higher stress responses than those seen in OFH chicks. Hence, to assess the relative benefit of on-farm hatching practices for practical applications, future research should compare physiological stress and cognitive responses between treatment in the rearing and laying phase and strive to replicate on-farm hatching procedures more closely to the envisioned future practices (e.g., integrating in ovo sexing for the OFH treatment).”*

- The newly hatched chicks were manually sexed - please provide more information on how this was done and how this may possibly have affected the chicks that remained in the experiment.

*We now added more details in the Material and methods section L. 91-95:*

*“Similar to OFH chicks, STAN chicks were vaccinated and manually sexed by examining their wing feathers for sex-specific patterns (this was however conducted at the hatchery facility). Although both OFH and TRAN chicks were manually sexed by the same company managing the post-hatch procedures (Prodavi SA, CH), we supervised the sorting of OFH chicks and encouraged the sexers to proceed gently, however no objective comparison was made of the handling procedures between treatment groups.”*

*In our discussion, we have now incorporated a section addressing limitations, particularly those associated with the early-life procedures. Given the similarity of the methods used for sexing both OFH and TRAN chicks (as now detailed in the method section) we believe that this process should not have affected the treatment differently, and therefore that it should not unduly affect our results.*

- I think the egg production data should in fact be possible to test in a more rigorous statistical manner. Although I appreciate that there were only five pens per treatment, a statistical evaluation would be helpful in order to validate the findings.

*We now added some statistical comparison between treatment for the egg-production data L.246-248:*

*“Then, to statistically assess treatment effect we fitted a nonlinear mixed-effects model with the ‘nlme’ package with treatment as fixed effect and pen identity as random effect on all three parameters (a, m, s).”*

*We also now analysed daily mortality data as suggested by reviewer 3.*

- The differences between treatment groups in KBF are surprising (to me at least) and I think the authors should consider the possibility that the early treatment in the OFH may have contributed (egg transport, sexing, vaccination).

*Thank you for the suggestion. While we certainly agree that some aversive procedures experienced by the OFH treatment may have contributed to the severity of KBF, we believe it is not the most likely explanation to explain the differences observed between treatment in the severity of KBF.* The reason for this is that *both OFH and TRAN chicks were sexed and vaccinated at approximately the same time and using same method, so we believe these processes would not explain the observed differences between the treatment. More generally, we designed the experiment with the specific intention of ensuring that all hatchery-related procedures should be perceived as less aversive, or when not possible, equivalently, for the OFH treatment compared to the TRAN treatment. We hope to have clarified these aspect in the new section “Limitation”, as copied in the response of your first comment.*

- In general, this is a commendable paper that should be published after the authors have considered my comments. However, it should also be noted that until in-ovo sexing is a feasible standard method, the differences between the hatchery treatments and the OFH may not be very large from the perspective of animal welfare.

*Thank you for this interesting point, we have adapted the sentence that was mentioning the in ovo sexing accordingly L.451-455:*

*“**Until in ovo sexing becomes practical at commercial scale, differences between hatchery and on-farm treatments may not have practical effects relevant to animal welfare. However, with the advancement of in ovo sexing techniques* [5]*, it is possible that on-farm hatching practices will become a standard method in the future. Therefore, it is important to compare both animal welfare and productivity between commercial hatchery and on-farm treatments within commercially relevant settings.”*

## **Reviewer 2**

Respected Authors,
The idea is very well conceptualized, and manuscript is written well, however, at this position I suggest some corrections.
I have mentioned all my suggestions/corrections in attached file, kindly follow the instructions.

*Many thanks for the positive appreciation and comments, especially those on the study design which was lacking important details. We hope to have clarified each of your comments in the below point-by-point responses. We copied pasted each of your comment you made on a separate document to provide more clear answers.*

 Ll.30.31: to which specie? please mention

*We now adjusted the text accordingly L.32-35:*

*“Exposure to less stressful environmental conditions during development may have a positive impact on individual fitness later on, a phenomenon called “silver-spoon” effect* [6] *(for a review on birds and mammals, see [5]).”*

L.61: check spellings

*We have gone over the spelling in this line and don’t see any issues. We have kept the language.*

L.71 on the title “Study design”: which experimental design was followed?

*Many aspects in our design were similar to a previous study conducted in our group on layer chicks* [1]*. We have now added text accordingly on L.84:*

*“Our methodology is similar to a previous study conducted on layer chicks* [1]*.”*

L.73: on the “3,300 eggs”: how sample size was determined?

*This sample size was determined to ensure that we would have enough healthy female chicks to fill two rearing pens (i.e. have 1’200 chicks), while accounting for the female-male ratio and the hatching rate. We therefore indeed planned a relatively a high number of eggs per treatment, but we also planned to return all extra chicks (male and female) to the hatchery for selling.*

L. 75 : give transportation details: ventilation during transportation or environment

We gave all details that we were given by the company, and unfortunately do not have details about the ventilation during transportation. All information we have about transportation is now written in the text L. 88-90:

“Transportation took place in a commercial vehicle over a duration of eight hours, during which a consistent environmental temperature around 28 °C was maintained.”

L.79 : add graph of egg shell temperature for better understanding.

*Thank you for the suggestion, we have now added the graph to the supporting information and referenced it on that line (now L.83):*

L.80 : on the “humidity above 30% » : why 30 % any specific reason?

*This was chosen based on a previous study conducted in our group on layer chicks* [1], which *was also based on the guidelines provided by Vencomatic, the company that supplied related equipment. We now added the reference in the main text.*

L.81 : on the « were manually sexed » : through which method?

*We agree more details is needed, and hope to have clarified that important aspect on L. 91-95:*

*“Similar to OFH chicks, STAN chicks were vaccinated and manually sexed by examining their wing feathers for sex-specific patterns (this was however conducted at the hatchery facility). Although both OFH and TRAN chicks were manually sexed by the same company managing the post-hatch procedures (Prodavi SA, CH), we supervised the sorting of OFH chicks and encouraged the sexers to proceed gently, however no objective comparison was made of the handling procedures between treatment groups.”*

L.82: on the “1,200 chicks” from this sentence: “On the same day, 1,200 chicks from the commercial hatchery were transported to the rearing barn as part of the STAN treatment.”: reviewers’ comment: average weight and SD

*Unfortunately, we do not have any information on the chick’s average weight and SD.*

L.96: stocking density? L.100 : Bird

*We now moved the information on bird density on L.96 (which is now L.105) and called it bird density, instead of animal density as suggested in the comment on L.100.*

L.133: better to add ethogram of behavioural repertoire

*Thank you for the suggestion. We considered this option but would prefer not doing so. Typically, an ethogram is employed to catalogue species-specific behaviours in order to describe the characteristics and function of each behaviour. However, the behaviors under consideration in this study exclusively pertain to spatial behavior and may not inherently possess specific functions in the traditional sense. Consequently, we believe that employing an ethogram in this context might not provide much benefit. Furthermore, considering the length of our manuscript, we would prefer not adding another Table.*

L.145 : on « animals » the reviewers wrote “bird”

*We replaced the word accordingly.*

L.149: add mathematical models for better understanding of data analysis

*Thank you for the suggestion. We gave an equation for the egg-production but believe that for the spatial and welfare variables for which we used generalized linear mixed-effect models, it is not required. Mathematical models for widely used statistics, such as generalized linear mixed-effect models, are often not provided in text, especially if all necessary information to replicate the analysis is within the text. We would greatly prefer not to provide the mathematical model for this specific section.*

*L. 235: in the table of the welfare indicators, the reviewers commented: “SD is not reliable estimate add standard error of means”*

*Our table refers to the observed raw values, and thus, standard deviation seems an adequate value to include. Standard errors are often used or inferential statistics, so we now added Table S2 in the supplementary information with the estimated marginal means and associated confidence intervals. Thus, we kept the table as is in the main manuscript but clarified the caption to increase clarity. If further changes are needed, we could add the estimates marginal means* (±SE) for the KBF severity (as the only welfare indicator with treatment difference) *in the main manuscript*:

*“*More specifically, the estimated marginal means (±SE) at the different day of age (DOA) was as follows: 215 DOA: 13.20 ±1.58 TRAN vs 17.70 ±1.56 OFH, 243 DOA: 16.70 ±1.56 TRAN vs 21.30 ±1.54 OFH, 313 DOA: 25.10 ±1.56 TRAN vs 29.60 ±1.54 OFH, 417 DOA: 35.00 ±1.58 TRAN vs 39.50 ±1.56 OFH (similar information for the other welfare indicators are presented in S2 Table).*”*

*L. 298: highlighting the section “discussion” the reviewer commented: “add some more logical reasoning of results”*

*We have now added additional language in the discussion that we believe adds more reasoning to our results. We hope this suffices.*

## ****Reviewer 3****

This is an interesting study that shows that on-farm hatching does not necessarily always result in improved welfare for the laying hens for the rest of their lives. In fact, it seems like traditionally hatched birds that have experienced transport in early life cope better with the move from rearing barn to laying barn. It is nice to see results honestly presented, even though I assume the authors started from the hypothesis that on-farm hatching would be beneficial (given the rest of the literature).

I don't have any major issues with this paper. The results seem clear, as do the methods. I do have a few requests for clarification, though, and a (very) few editing suggestions.

*We thank you for your positive appreciation on the manuscript and helpful comments. We hope to have clarified all of your points in the below point-by-point responses, and believe these have contributed to improving the manuscript, particularly your suggestion about the mortality data.*

1) Could the authors please explain a bit better the implications of the fact that some birds were killed and then replaced by other animals? Does this mean that the sample of OFH and STAN birds at different time points were partially overlapping, but not identical groups of birds? Does that have any implications for the stats?

*Indeed, over time we do not have the exact same group of hens. At five time points during the laying phase 16 hens were killed (8 OFH, 8 TRAN) and replaced with new hens arbitrarily chosen. In other words, we had approximately half of the hens that stayed throughout the entire study, whereas the other half were killed at some time point and replaced. Because this was done similarly for both OFH and TRAN animals (i.e., the same number of animals on the same day) and that we are here analysing the difference between the two treatments, we don’t think it could have affected the results. However, we remain open to discuss this in the new “limitation” paragraph at the end of the discussion.*

2) In the statistics, often different data structures were chosen for different variables. Please explain how you decided on each of these structures and why.

*We now have clarified why we had to use different data distribution (basically, to meet model assumptions) when explanation was missing, and most importantly we analysed the sigmoid curves fitted to the daily average number of egg per live hens until the end of production and not only until day 60, so that the data structure better coincide with those of the spatial behaviour. As requested by another reviewer, we also tested the treatment effect statistically in this analysis.*

3) Given the recently presented results on the difference between KBF high on the keel (typically on the outside, and from collisions) vs. low on the keel (typically on the inside and due to egg laying), do the authors have any information on whether the difference between OFH birds and STAN birds was mostly due to "collision breaks" or to "egg laying breaks"? The discussion suggests they believe collisions are more to blame due to inexperience with moving around the aviary, but it would be good to see the other discussed.

*This is indeed a very interesting point that we have now integrated into the discussion* *on L.381-387:*

*“More recent evidence suggested that factors related to egg production, including internal pressure during the egg-laying process* [7] *and an early onset of lay* [8]*, could favour KBF. We found no evidence of treatment effect in the average number of egg per live hens, nor did we observe treatment effect on the timing of the onset of lay. In fact, a previous study showed that on-farm hatched hens had a slower onset of egg-laying than hens hatched in commercial hatchery at 15-20 weeks of age* [9]*. Hence, while an early onset of laying may not explain the treatment effect on KBF observed in our study, it is plausible that other factors related to the internal pressure during the egg-laying process could be operating in the current effort.”*

4) I don't quite follow the reasoning on line 395 about counterbalancing. The study did not really assess HPA axis activity, so what are the authors trying to explain away here, then?

*Thank you for raising that point, we were indeed not clear enough. We clarified the paragraph, and replaced “….have counterbalanced potential alterations of the HPA axis” by “have triggered the HPA axis of OFH hens to reach conditions resembling that of the STAN hens”. Indeed, as you mentioned, we did not assess the HPA-axis activity. As early life stressors are known to affect the HPA-axis, we are relating this important part of literature to our study, because we believe that it is an important aspect to consider in future studies. We hope to have now clarified the text.*

5) The authors don't report this, but I wonder if there was any effect of OFH on mortality in the laying barn (and therefore on total egg production, rather than per-bird production).

*Thank you again for raising this point. We now analysed the daily mortality (using survival analysis), were we found no statistical difference between treatment:*

*We therefore also replaced all sub-section names “egg-production data” by “Production traits”.*

a. line 140 "between 2 and 8 o'clock" instead of "2 and 8 hour"? Or do you mean between 2 and 8 hours after lights came on?

*Thank you, we now changed it according to your first proposition: between 02:00h and 08:00h, as it is indeed the time of the day, and not the number of hours after lights came on.*

b. line 153: "KBF severity and feather damage" instead of "KBF severity the feather damage"?

*Sorry for this typo, we now changed it accordingly, thank you for pointing this out to us.*

c. line 234: although what you say is not wrong, the fact that red is left of blue in the figure makes it more logical to write "Red and blue colours represent the treatment groups OFH and STAN respectively", as they are then in the same order as in the figure.

*Indeed, we changed it accordingly.*

d. line 238: "Model coefficients" instead of "Models' coefficients"

*Thank you again for highlighting these typos.*

e. line 239: "To help further interpret" instead of "To help further interpreting"

*We changed accordingly.*

f. line 342-343: please clarify the phrase "potential decreased weight gained" - I get confused about what goes up and what goes down.

*We now changed “because* of the potential decreased weight gain due to early life stress” by “…because it is possible that animals may gain less weight due to early life stress”. *We hope this clarifies the language.*

**References**

[1]	Witjes VL, Bruckmaier RM, Gebhardt-Henrich SG, Toscano MJ. Effects of on-farm hatching on short term stress indicators, weight gain, and cognitive ability in layer chicks. Appl Anim Behav Sci 2022:105692. https://doi.org/10.1016/J.APPLANIM.2022.105692.

[2]	De Jong IC, Van Riel J, Bracke MBM, Brand H van den. A “meta-analysis” of effects of post-hatch food and water deprivation on development, performance and welfare of chickens. PLoS One 2017;12:e0189350. https://doi.org/10.1371/JOURNAL.PONE.0189350.

[3]	Mitchell MA. Chick transport and welfare. Avian Biol Res 2009;2:99–105. https://doi.org/10.3184/175815509X431894/ASSET/175815509X431894.FP.PNG_V03.

[4]	Nordquist RE, Vernooij JCM, Dull CL, Pascual A, van der Linde G, Goerlich VC. The effects of transport of 18-day old hatching eggs on physiology and behaviour of slow growing broiler chicken. Appl Anim Behav Sci 2022;257:105789. https://doi.org/10.1016/J.APPLANIM.2022.105789.

[5]	Krautwald-Junghanns ME, Cramer K, Fischer B, Förster A, Galli R, Kremer F, et al. Current approaches to avoid the culling of day-old male chicks in the layer industry, with special reference to spectroscopic methods. Poult Sci 2018;97:749–57. https://doi.org/10.3382/PS/PEX389.

[6]	Grafen A. On the uses of data on lifetime reproductive success. In: T. H. Clutton-Brock, editor. Reproductive success, vol. 363, 1988, p. 454–71.

[7]	Thøfner I, Hougen HP, Villa C, Lynnerup N, Christensen JP. Pathological characterization of keel bone fractures in laying hens does not support external trauma as the underlying cause. PLoS One 2020;15:e0229735. https://doi.org/10.1371/JOURNAL.PONE.0229735.

[8]	Gebhardt-Henrich SG, Fröhlich EKF. Early Onset of Laying and Bumblefoot Favor Keel Bone Fractures. Animals 2015;5:1192–206. https://doi.org/10.3390/ANI5040406.

[9]	Hedlund L, Whittle R, Jensen P. Effects of commercial hatchery processing on short- and long-term stress responses in laying hens. Sci Rep 2019;9:1–10. https://doi.org/10.1038/s41598-019-38817-y.
